# Comparative genomics of muskmelon reveals a potential role for retrotransposons in the modification of gene expression

**DOI:** 10.1038/s42003-020-01172-0

**Published:** 2020-08-13

**Authors:** Ryoichi Yano, Tohru Ariizumi, Satoko Nonaka, Yoichi Kawazu, Silin Zhong, Lukas Mueller, James J. Giovannoni, Jocelyn K. C. Rose, Hiroshi Ezura

**Affiliations:** 1 Faculty of Life and Environmental Sciences, University of Tsukuba, 1-1-1 Tennodai, Tsukuba, Ibaraki 305-8572 Japan; 2 Advanced Analysis Center, National Agriculture and Food Research Organization (NARO), 2-1-2 Kannondai, Tsukuba, Ibaraki 305-8518 Japan; 3 JST PRESTO, 4-1-8, Honcho, Kawaguchi, Saitama 332-0012 Japan; 4 Tsukuba Plant Innovation Research Center (T-PIRC), University of Tsukuba, 1-1-1 Tennodai, Tsukuba, Ibaraki 305-8572 Japan; 5 Institute of Vegetable and Floriculture Science, National Agriculture and Food Research Organization (NARO), Tsu, Mie 514-2392 Japan; 6 Boyce Thompson Institute for Plant Research, Ithaca, NY 14853 USA; 7 State Key Laboratory of Agrobiotechnology, School of Life Sciences, The Chinese University of Hong Kong, Hong Kong, China; 8 U.S. Department of Agriculture/Agriculture Research Service, Robert W. Holley Centre for Agriculture and Health, Ithaca, NY 14853 USA; 9 Plant Biology Section, School of Integrative Plant Science, Cornell University, Ithaca, NY 14853 USA

**Keywords:** Natural variation in plants, Genome duplication, Comparative genomics, Molecular evolution

## Abstract

Melon exhibits substantial natural variation especially in fruit ripening physiology, including both climacteric (ethylene-producing) and non-climacteric types. However, genomic mechanisms underlying such variation are not yet fully understood. Here, we report an Oxford Nanopore-based high-grade genome reference in the semi-climacteric cultivar Harukei-3 (378 Mb + 33,829 protein-coding genes), with an update of tissue-wide RNA-seq atlas in the Melonet-DB database. Comparison between Harukei-3 and DHL92, the first published melon genome, enabled identification of 24,758 one-to-one orthologue gene pairs, whereas others were candidates of copy number variation or presence/absence polymorphisms (PAPs). Further comparison based on 10 melon genome assemblies identified genome-wide PAPs of 415 retrotransposon Gag-like sequences. Of these, 160 showed fruit ripening-inducible expression, with 59.4% of the neighboring genes showing similar expression patterns (*r* > 0.8). Our results suggest that retrotransposons contributed to the modification of gene expression during diversification of melon genomes, and may affect fruit ripening-inducible gene expression.

## Abstract (web summary)

Ryoichi Yano et al. report a Nanopore-based reference genome assembly of muskmelon—a fruit known for its many cultivated varieties, including cantaloupe and honeydew—using the Japanese Harukei-3 cultivar. They identify structural genetic variation by comparing the reference to several melon genome assemblies and investigate tissue-wide gene expression patterns by RNA sequencing.

## Introduction

Melon (*Cucumis melo* L.) is one of the most economically important fruit crops in the world and is a source of vitamins, minerals, and other health-promoting substances. It is thought to have originally diversified in India and Asia and is known to exhibit very wide natural variation, especially in fruit phenotypes^1–3^. At least 19 horticultural subgroups and six major groups of melon have been identified. A particularly notable feature of melon is the coexistence of both climacteric (ethylene-producing and showing a burst in respiration at the onset of ripening) and non-climacteric fruit types^4–6^. For example, the French cultivar “Vedrantais”, belonging to the subgroup var. *cantalupensis*, is well-known example of a climacteric melon, whereas melons of the subgroup var. *inodorus* (e.g., the American cultivars “Honey dew” and Spanish “Piel de Sapo”) are non-climacteric. The molecular mechanism of ethylene production has been intensively studied in melon, given the importance of this hormone in regulating climacteric fruit-ripening traits such as shelf life, which is of considerable economic importance^7–11^.

The melon genome comprises 12 chromosomes, and its genome size was estimated to be ~454 Mb based on the nuclear DNA content^12^. This is larger than the genomes of other cucurbit plants such as *Cucumis sativus* (7 chromosomes, 367 Mb) and *Citrullus lanatus* (11 chromosomes, 425 Mb). The first reported whole genome sequence of melon was that of the experimental line designated DHL92^13^, which was originally derived from a cross between the non-climacteric “Piel de Sapo” (subsp. *melo* var. *inodorus*) and the Korean landrace “Songwhan Charmi” (subsp. *agrestis* var. *chinensis*). A genomic DNA sequence of 417 Mb was published in the latest version of the DHL92 genome reference (CM3.6.1), of which 337 or 79.6 Mb was the actual nucleotide sequence or ambiguous bases (e.g., NNN), respectively^14^. In addition, 29,980 protein-coding genes have been reported in the genome annotation CM4.0^14^. The DHL92 genome reference has been utilized for supporting transcriptome analyses as well as quantitative trait loci (QTL) studies of important agricultural traits, including fruit ripening, fruit morphology, and disease resistance^5,11,15–19^. With decreasing costs of whole genome sequencing, several melon accessions have been sequenced and characterized using the Illumina short read next-generation sequence (NGS) platform^20,21^. However, third generation sequencing technologies (e.g., PacBio RSII/sequel and Oxford Nanopore Technology [ONT]), implemented with single molecule sequencing that can generate long reads (e.g. >10 kb) emerged as an alternative approach. Many plant genomes have been assembled and/or re-evaluated using such newer DNA sequencers^22–28^, including the genome of the Chinese *inodorus* melon cultivar Payzawat, which was sequenced using a PacBio RSII platform^29^. In addition, such approaches can provide insights into genome structural variation, as was demonstrated in *Solanum lycopersicum* using ONT ultra-long sequencing technology^22^.

At present, little is known about genomic structural variation, especially copy number variation (CNV) and presence/absence polymorphism (PAP) in the genomes of melon subgroups. In this current study, we assembled the whole genome sequence of the semi-climacteric Japanese cultivar “Earl’s Favorite Harukei-3” (Harukei-3; var. *reticulatus*) by coupling ultra-long ONT sequencing (R9.4.1 + R10 flow cells) with Bionano optical mapping and Illumina mate pair sequencing. This melon shows moderate climacteric ripening behavior, although the rate of ripening is less than that of the well-studied cultivar Charentais (var. *cantalupensis*)^4^. ONT RNA-seq-based gene prediction coupled with other methods, such as ab initio prediction, also identified 33,829 protein-coding genes whose protein BUSCO benchmark value was 1372 (95.3%). We also expanded our tissue-wide transcriptome (RNA-seq) dataset of the Melonet-DB (https://melonet-db.dna.affrc.go.jp/ or https://gene.melonet-db.jp) by adding RNA-seq samples of ethylene-producing ripening fruit. In addition, the genomes of seven more melon accessions were sequenced and assembled by ONT at the contig level to conduct the assembly-based PAP analysis of retrotransposon Gag-like sequences. Based on a combination of comparative genomics and comprehensive transcriptome analysis, we suggest that retrotransposons played a role in the modification of gene expression as well as evolution of fruit-ripening-inducible gene expression during diversification of melon genomes.

## Results

### Genome assembly and comparative genomics of Harukei-3 melon

**Fig. 1:**
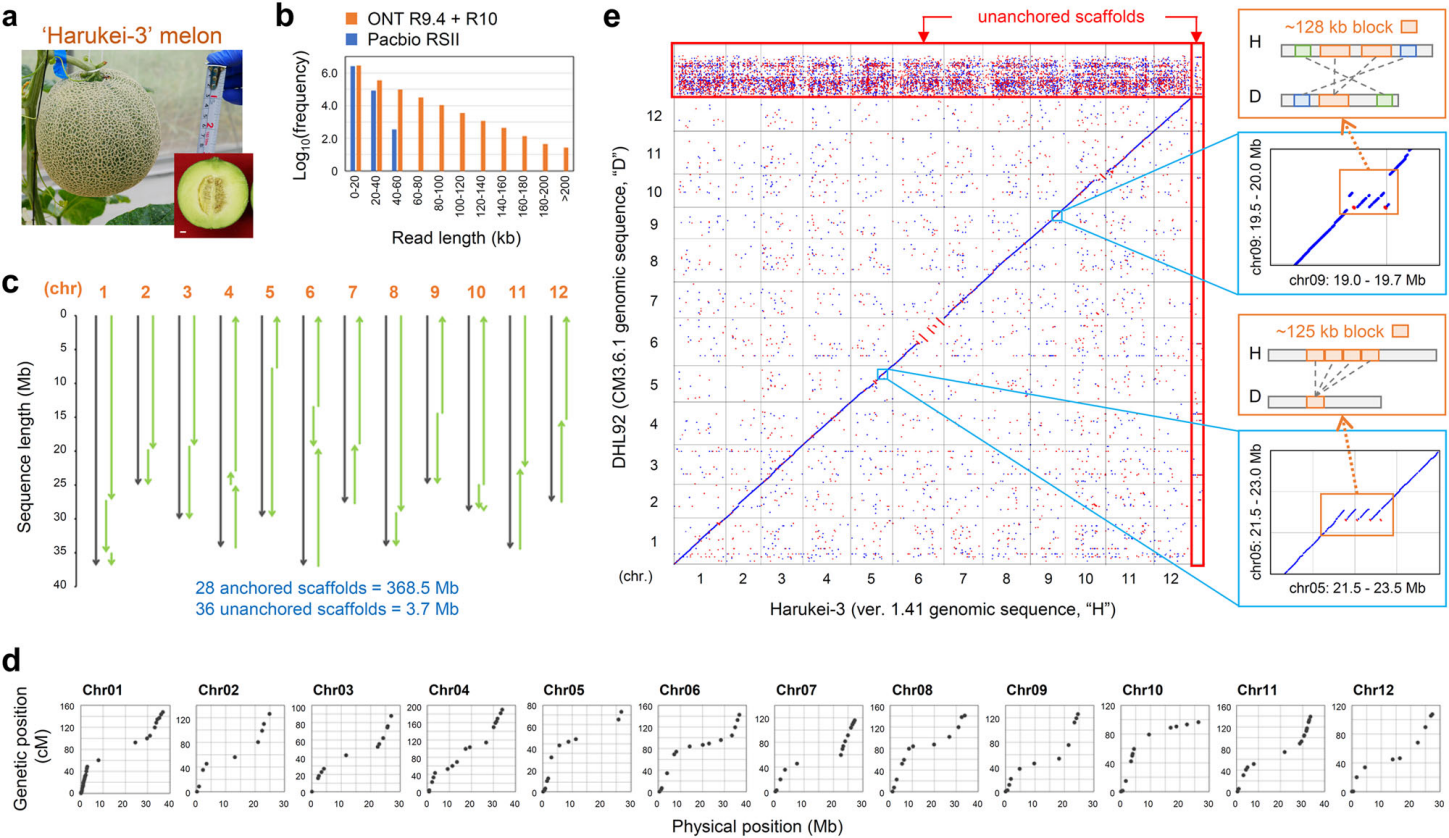
Whole genome assembly of Harukei-3 melon. **a** Harukei-3 melon fruit. **b** Histograms of sequenced reads in Oxford Nanopore technology (ONT, R9.4.1 and R10 flow cells) or PacBio RSII. Ultra-long reads with >60 kb are present only in ONT dataset. Reads with ≥5 kb were used for de novo assembly (for detailed procedure see Supplementary Fig. 2). **c** Construction of the chromosome-scale pseudomolecule in Harukei-3. **d** Comparison between linkage map and assembled pseudomolecule. Linkage maps were obtained in the genetic population derived from Harukei-3 and I-10 accessions^16^. Physical and genetic positions of 167 markers are shown. **e** Genomic alignment between Harukei-3 and DHL92 genomes. Right panels show examples of large genomic block duplication (>120 kb) that are present (or assembled) in the Harukei-3 genome. Blue and red dots indicate that DNA is aligned in forward or reverse directions, respectively. Unanchored sequences are indicated by red rectangles.

Melon is usually described as producing sweet fruit; however, Harukei-3 produces considerably sweeter fruit than other melon accessions if it is grown in the appropriate seasons (Supplementary Fig. 1). Indeed, the Japanese word “Harukei” means a line suitable for growing in the spring. As a consequence of its taste and attractive appearance (Fig. 1a), Harukei-3 has been used for a long time in Japan as a standard melon to breed high-grade muskmelon. To investigate the genome structure of Harukei-3, and to obtain functional gene information for future genetic studies and breeding, we assembled its genome sequence by combining ONT ultra-long sequencing (R9.4.1 and R10), Bionano optical map, Illumina Hiseq, mate pair, and linkage map information (summarized in Supplementary Fig. 2). The ONT platform yielded more long reads than did PacBio RSII, and these ONT long reads were used to generate a contig assembly with *N*_50_ = 8.6 Mb that was 10 times higher than that of the PacBio contig (Fig. 1b and Supplementary Fig. 2). After scaffolding with the Bionano map and mate pair data, we obtained 80 ONT-based genomic scaffolds with an *N*_50_ = 17.5 Mb. We also assembled the genomic scaffold based on PacBio RSII using the same procedure (*N*_50_ = 11.4 Mb). Using PacBio-based scaffolds as a hint to modify the ONT-based scaffolds, we obtained 66 genomic scaffolds with a physical gap number of 92 and an *N*_50_ = 18.9 Mb. Finally, 12 chromosome sequences were constructed using 28 scaffolds based on linkage map information (Fig. 1c and Table 1; Harukei-3 ver. 1.41 pseudomolecule). The chromosomal sequence lengths without ambiguous bases (e.g., NNN) from Harukei-3 (366.7 Mb) were much longer than those from DHL92 (318.2 Mb), reflecting the lower number of physical gaps in the Harukei-3 genome sequence (Table 1). The chloroplast genome was likely to be entirely assembled because we obtained two kinds of contigs with sequence lengths of 155 and 156 kb (Table 1). When the Harukei-3 genomic sequence was compared with the linkage map of Harukei-3^16^, we observed complete co-linearity between physical positions and linkage positions (Fig. 1d), indicating that the Harukei-3 genome sequence was correctly assembled at a chromosome-scale. In contrast, when it was compared with the linkage map of other accessions or sources^13,30,31^, the physical position did not match the linkage map position of some markers (Supplementary Fig. 3), suggesting chromosome-level structural differences between the genomes of melon accessions.

**Table 1:** Summary of assembled sequence lengths and predicted genes in the Harukei-3 genome. DNA sequence lengths of each chromosome and unanchored sequence are compared between Harukei-3 ver. 1.41 reference (left) and DHL92 CM3.6.1/4.0 reference (right). In Harukei-3, organelle genomes are assembled as one scaffold.

Harukei-3 (ver. 1.41)	Total (kb)	Determined (kb)	Undetermined (“NNN”, kb)	Gap	Gene count	DHL92 (CM3.6.1/4.0)	Total (kb)	Determined (kb)	Undetermined (“NNN”, kb)	Gap	Gene count
chr01	36,940	36,524	416	16	3703	chr01	37,038	31,291	5747	2273	2807
chr02	24,998	24,882	116	4	2493	chr02	27,065	23,792	3273	1536	2064
chr03	30,037	29,911	126	2	2798	chr03	31,667	26,381	5286	1887	2229
chr04	34,190	33,842	348	9	3660	chr04	34,318	29,761	4557	2099	2833
chr05	29,725	29,488	236	10	2603	chr05	29,324	24,896	4428	1864	2021
chr06	36,910	36,514	396	12	3580	chr06	38,297	31,984	6313	2260	2847
chr07	27,657	27,296	360	7	2801	chr07	28,958	24,137	4822	1594	2235
chr08	34,052	33,922	130	4	3243	chr08	34,765	28,980	5786	2079	2569
chr09	24,854	24,615	239	6	2588	chr09	25,243	21,670	3573	1577	2016
chr10	28,914	28,643	271	6	2617	chr10	26,664	22,706	3958	1540	1801
chr11	34,404	34,011	393	7	3307	chr11	34,457	28,852	5605	2001	2535
chr12	27,488	26,893	595	11	2701	chr12	27,564	23,699	3865	1735	2056
Total	370,168	366,542	3625	94	36,094	Total	375,360	318,148	57,212	22,445	28,013
Unanchored	7215	2045	5170	43	645	Unanchored	41,642	19,177	22,465	22,207	1967
Mitochondria	3273	3249	24	5	317						
Chloroplast1	156	156	0	0	66						
Chloroplast2	155	155	0	0	82						
Chloroplast3	96	96	0	0	50						

We conducted genomic alignment of Harukei-3 and DHL92 and while most of the alignment showed co-linearity, a large number of small genomic sequences were observed that might have resulted from translocation across the chromosomes between the two genomes (Fig. 1e). Additionally, the Harukei-3 assembly revealed the duplication of a large genomic block (>120 kb block repeat on chromosome 5 or 9), but this was not apparent in the DHL92 genome. We previously constructed the Harukei-3 genomic pseudomolecule based on PacBio RSII data; however, this version of the genome assembly did not resolve the large genomic block duplication (Supplementary Fig. 4), underlining the value of the ONT ultra-long reads in resolving the genomic structure. We also conducted a genomic alignment between Harukei-3 and the recently published genome of the *inodorus* melon, Payzawat, which was assembled based on PacBio data^29^. This revealed a large genomic block duplication on chromosome 5 of Harukei-3 that was absent, or not assembled, in the Payzawat genome (Supplementary Fig. 5). Other large genomic blocks were also absent in the Payzawat genome (e.g., the upper part of chromosome 8).

To compare the genomes based on gene information, we newly predicted genes in the Harukei-3 genomic sequence. We mainly used ONT RNA-seq for this purpose as ONT RNA-seq analysis can yield nearly full-length sequence mRNA molecules (summarized in Supplementary Figs. 6,7). By combining ab initio prediction (e.g., AUGUSTUS) and short read-based prediction (e.g., Braker2) as supplementary methods, we identified 33,829 protein-coding genes in the Harukei-3 genome (33,314 nuclear genes + 515 organelle genes). Both protein BUSCO benchmark analysis^32^ (ver. 3.0) and an InterProScan search^33^ indicated that the Harukei-3 genome annotation represents a highly comprehensive dataset of plant protein-coding genes compared with published cucurbit genomes^26–28,34–40^ (complete BUSCO = 1372 [95.3%] and InterPro ID count = 5607; Table 2 and Supplementary Data 1). In addition, we observed that our ONT-based gene prediction method gave accurate insights into exon-intron gene structure, whereas other methods (e.g., AUGUSTUS and Braker2) gave incorrect assessments of structure in some genes (Supplementary Fig. 8). We also searched for repetitive elements in the Harukei-3 genome (e.g., simple sequence repeats and DNA/RNA transposons). In total, 211 Mb of the Harukei-3 genomic sequence was found to correspond to repetitive elements (Supplementary Data 2).

**Table 2:** Harukei-3 genome annotation contains comprehensive set of plant protein-coding genes. Completeness of protein-coding gene dataset was assessed by protein BUSCO benchmark ver. 3.0^32^ and InterProScan^33^, and the BUSCO benchmark scores and the counts of identified GO and InterPro ID are compared between Harukei-3 and other genomes including 12 published cucurbit genomes.

	Protein-coding gene	Protein BUSCO benchmark (ver. 3.0)			GO/InterProScan ID count			
Genome reference (annotation version)		Complete	Fragmented	Missing	GO: BP	GO: MF	GO: CC	InterPro ID
*Cucumis melo* (var. *reticulatus*, Harukei-3 ver. 1.41)	33,829	1372 (95.3%)	16	52	638	845	200	5607
*Cucumis melo* (var. *inodorus x conomon*, DHL92 CM4.0)^14^	29,980	1257 (87.3%)	68	115	625	833	198	5533
*Cucumis melo* (var. *inodorus x conomon*, DHL92 CM3.5)^13^	27,427	1175 (81.6%)	85	180	631	839	196	5515
*Lagenaria siceraria* (USVL1VR-Ls)^34^	22,472	1233 (85.6%)	85	122	615	830	192	5381
*Cucumis sativus* (PI183967)^39^	22,790	1309 (90.9%)	45	86	626	836	198	5537
*Cucumis sativus* (Gy14, v1)^40^	21,503	1286 (89.3%)	53	101	632	847	201	5511
*Cucumis sativus* (Chineese Long, v3)^26^	24,317	1314 (91.3%)	38	88	628	841	200	5570
*Cucurbita argyrosperma*^35^	28,298	1268 (88.1%)	75	97	618	828	194	5426
*Cucurbita moschata* (Rifu ver. 1.1)^36^	32,205	1333 (92.6%)	37	70	627	841	200	5546
*Citrullus lanatus* (97103 ver. 2)^27^	22,596	1313 (91.2%)	40	87	623	836	199	5498
*Citrullus lanatus* (Charleston Gray, v2)^37^	22,545	1298 (90.1%)	44	98	611	823	196	5418
*Benincasa hispida* var. B227^28^	27,467	1321 (91.7%)	43	76	632	841	197	5505
*Cucurbita maxima* (Rimu ver. 1.1)^36^	32,076	1338 (92.9%)	31	71	632	841	200	5551
*Cucurbita pepo*^38^	27,868	1232 (85.6%)	55	153	610	823	195	5411
*Arabidopsis thaliana* (Col-0 TAIR10)	27,416	1432 (99.4%)	3	5	621	838	199	5618
*Solanum lycopersicum* (Heinz1706 ITAG4.0)^24^	34,075	1348 (93.6%)	45	47	636	853	200	5628

*BP* biological process, *MF* molecular function, *CC* cellular component

**Fig. 2:**
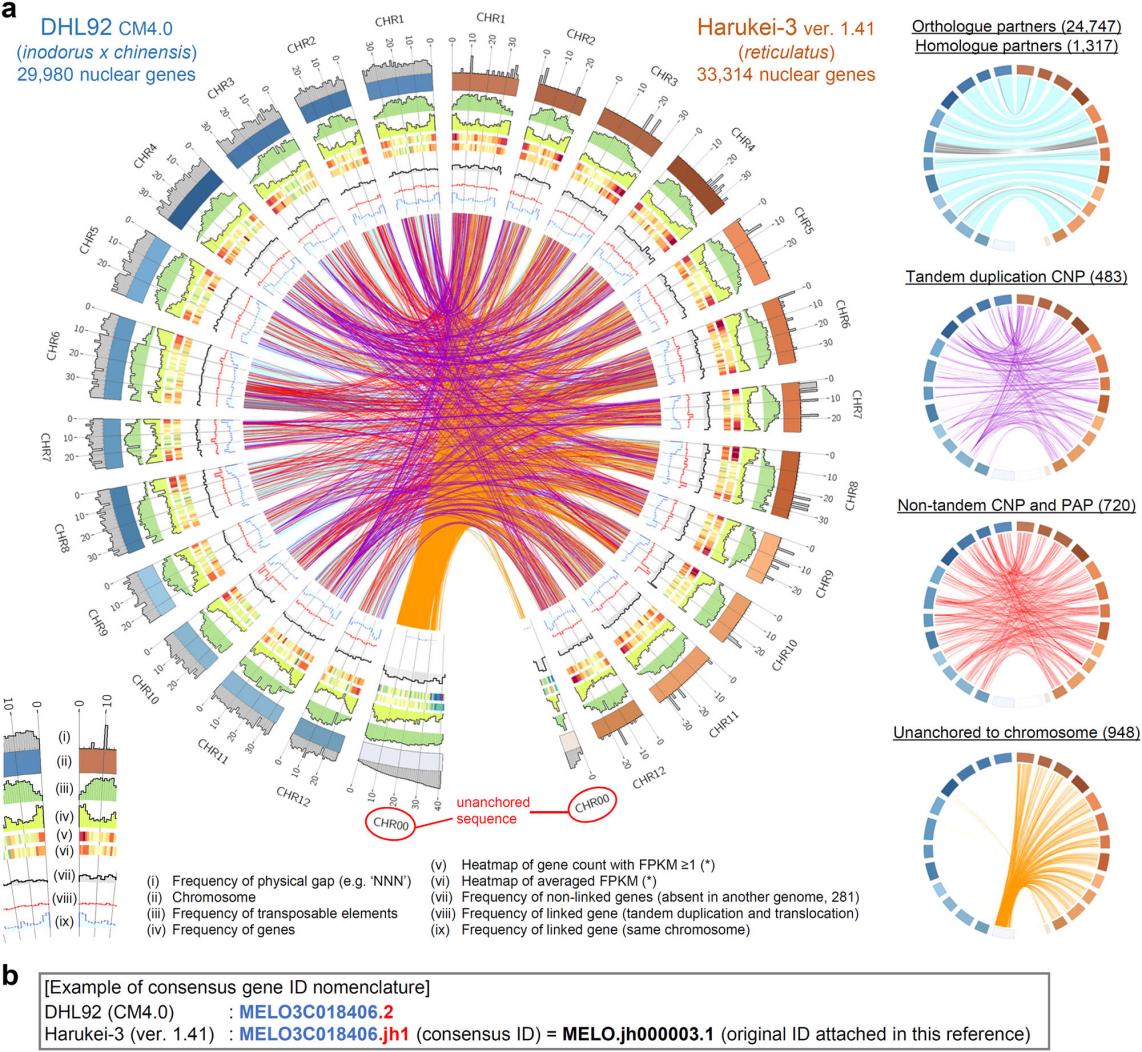
Comparison of Harukei-3 and DHL92 genome references. **a** A circos plot comparing Harukei-3 and DHL92 genomes. Frequencies of physical gap [i], transposable elements [iii], predicted genes [iv], genes absent in either genome [vii], candidate genes for copy number variation (CNV) and presence/absence polymorphism (PAP) [viii], orthologue and homologue partners [ix] are drawn in the plot together with the chromosome [ii], heatmaps of gene count with FPKM (Fragments Per Kilobase of exon per Million mapped fragments; gene expression levels) ≥ 0.1 [v] or averaged FPKM [vi] in the tissue-wide transcriptome dataset shown in Fig. 3. Orthologous or homologous gene partners are shown by links in the center of the plot (also shown in the right panels). One-to-one orthologous gene partners (24,747 links) or homologous partners (1317 links) are indicated by blue and gray lines in which blue line indicate that genes are located at the same direction while gray lines indicate the opposite direction. Purple, red, and orange links indicate 483 tandem duplication CNVs, 720 non-tandem CNVs and PAPs, and 948 genes that are unanchored in either genome, respectively. **b** Gene ID nomenclature in the Harukei-3 ver. 1.41 genome reference. Consensus gene ID that starts with “MELO3C” string are attached to 24,758 Harukei-3 genes that have one-to-one orthologues in DHL92 genome.

We next analyzed the gene partner relationship between Harukei-3 and DHL92 genomes based on bidirectional BLAST-n/p search^41^ and transcript alignment analysis with the BLAST-like alignment tool^42^ (Fig. 2a). Of the 33,314 nuclear genes of the Harukei-3 genome, 24,747 and 1317 genes showed one-to-one orthology or a homologous partner relationship, respectively, with DHL92 genes (Supplementary Data 3). We attached a consensus gene ID to the 24,747 orthologous genes of the Harukei-3 genome (a gene ID that starts with the “MELO3C” string and ends with the “.jh1” string) to help maintain a consistent gene nomenclature among melon genomes (Fig. 2b). Although it is generally difficult to compare reference genomes that are generated by different sequencing technologies, 1203 genes were identified as possible candidates for CNV and PAP (Fig. 2a, Supplementary Fig. 9a, Supplementary Data 4 and 5). Most of the unanchored DHL92 genes were also positioned on one of the 12 Harukei-3 chromosomes.

In addition to the assembly level genome comparison, we resequenced six melon accessions (Harukei-3, Honey dew [var. *inodorus*], Spicy [var. *cantalupensis*], Manshuu and Ougon-9 [var. *makuwa*], and JSS6 [*C. agrestis*, wild melon]) using Illumina Hiseq for comparisons with the three melon genome references (Harukei-3, DHL92, and Payzawat). While Honey dew and Spicy are American melon accessions, Manshuu and Ougon-9 belong to the Asian melon accession and JSS6 is a wild melon collected in Japan. Although the read alignment ratio was comparable between the three genome references, the Harukei-3 reference showed slightly better alignment ratios relative to the other two genome references in the resequencing data of Harukei-3 itself, Honey dew, and Spicy (Supplementary Fig. 10a). In contrast, the DHL92 reference showed better alignment ratios in comparison to the other two references in the case of *makuwa* and wild melon. Unlike the alignment ratio, the number of polymorphisms (single nucleotide polymorphisms (SNPs) and small insertions/deletions (Indels)) that were predicted to affect protein amino acid sequence was highly variable between the Harukei-3 and DHL92 references (Supplementary Fig. 10b). These results underlined the importance of using several genome references in the resequencing study.

### Co-expression analysis of fruit-ripening-inducible genes

**Fig. 3:**
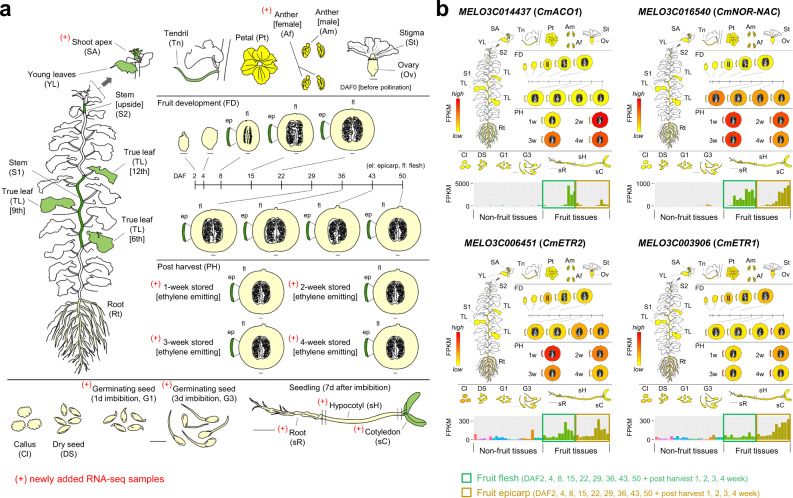
Tissue-wide transcriptome (RNA-seq) dataset in Harukei-3 melon. **a** A cartoon illustrating the updated tissue-wide RNA-seq dataset of Harukei-3 melon. RNA-seq of post-harvest ripening fruit (flesh: 4, epicarp: 4), shoot apex, female flower anther, imbibed seeds (1 and 3 days after imbibition), and 7-day seedlings (root, hypocotyl, cotyledon) were newly added to the previous dataset^18^. **b** Gene expression patterns of known fruit-ripening-related genes (*CmACO1*, *CmETR1* and *2*, *CmNOR-NAC*) in the updated tissue-wide transcriptome dataset. A new version of “Gene expression map viewer” in Melonet-DB (https://melonet-db.dna.affrc.go.jp/ap/mvw) was used to obtain the electro-fluorescent pictogram images.

**Fig. 4:**
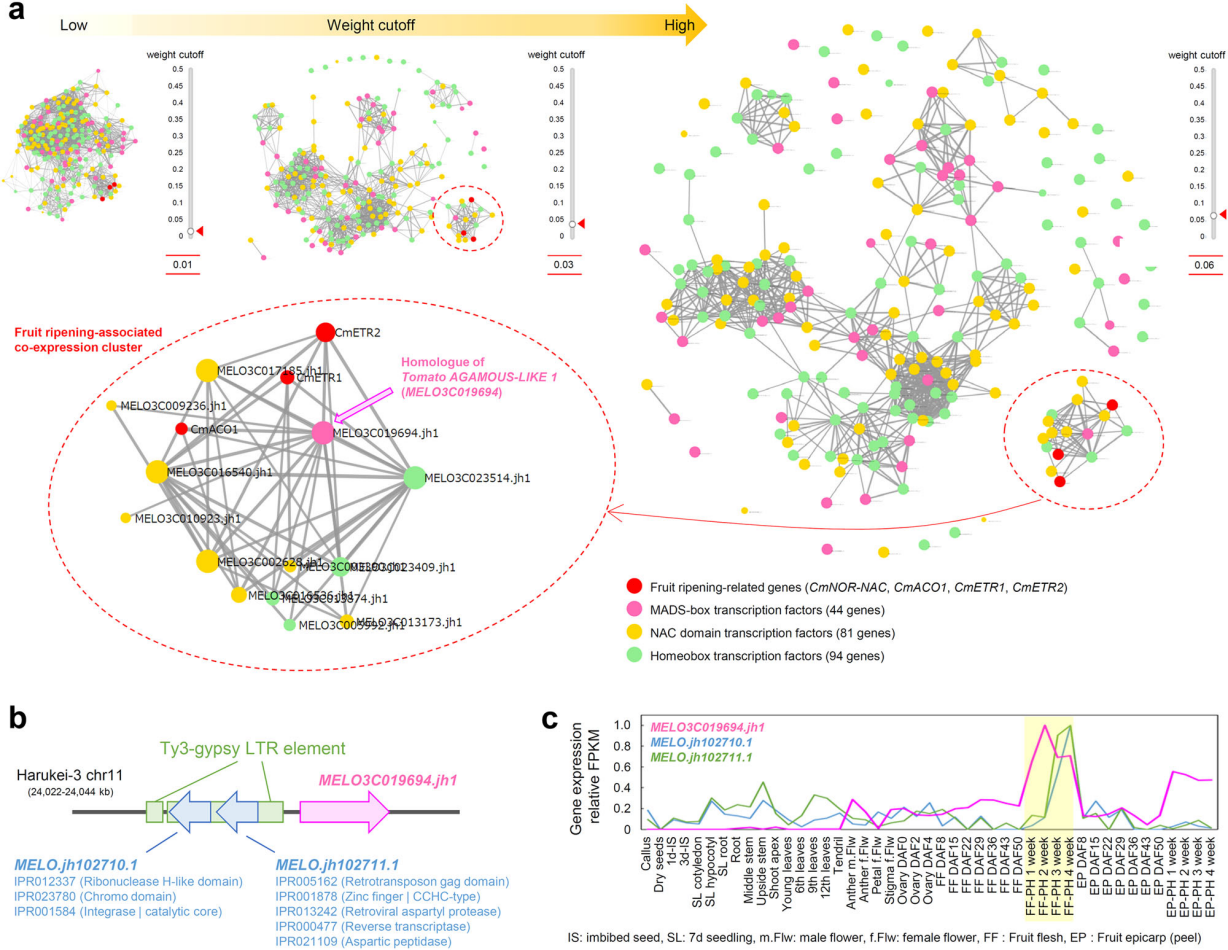
Identification of a long terminal repeat (LTR) retrotransposon in the promoter region of *MELO3C001864*, a potential core regulator of fruit ripening. **a** Co-expression analysis of four known fruit-ripening-related genes with 81 NAC domain, 90 homeobox, and 42 MADS-box transcription factors based on the updated tissue-wide transcriptome dataset. In the updated version of “Co-expression viewer” in the Melonet-DB (https://melonet-db.dna.affrc.go.jp/ap/mds), the weight cutoff value is changeable in a real-time manner with the slider bar function present in the interface window. At weight cutoff = 0.06, one co-expression cluster, including known fruit-ripening-related genes, is detached from other clusters (shown by dashed red circles). A melon homologue of *Tomato AGAMOUS-LIKE 1* (*TAGL1*), *MELO3C019694*, is positioned at the center of this fruit-ripening-associating co-expression cluster (indicated by pink-filled arrow). **b** Schematic illustration of the genomic region around *MELO3C019694*. Two long terminal repeat (LTR)/gypsy elements (1956 bp and 6941 bp) are identified by RepeatMasker in the promoter region of *MELO3C019694* in Harukei-3 genome (Supplementary Data 2). Around this region, two retrotransposon-related protein-coding sequences were located: *MELO.jh102710.1* and *MELO.jh102711.1*. **c** Tissue-wide gene expression patterns of *MELO3C019694* and neighboring retrotransposon-related sequences. Their expression levels were highest in post-harvest ripening fruits (indicated by a yellow box).

Since Harukei-3 fruit produce ethylene during ripening, we investigated ethylene-related gene expression in Harukei-3 and updated the tissue-wide melon RNA-seq transcriptome dataset of Yano et al.^18^ by adding data derived from ethylene-emitting ripe fruit (Fig. 3a). Alignment ratios of RNA-seq reads were much higher in Harukei-3 genome reference than in DHL92 probably because the RNA-seq data were obtained from Harukei-3 itself. We identified 27,687 Harukei-3 genes with Fragments Per Kilobase of exon per Million mapped fragment (FPKM) values ≥ 0.1 (Supplementary Fig. 11, Supplementary Data 6 and 7). Such high-sensitive detection of gene expression level enabled high-resolution co-expression analysis, and weighted genome-wide correlation network analysis^43^ (WGCNA) identified >60 co-expression clusters, including those specific to ripening fruit (Supplementary Fig. 12). We also updated the Melonet-DB web-application tools, “Gene expression map viewer” (https://melonet-db.dna.affrc.go.jp/ap/mvw) and “Co-expression viewer” (https://melonet-db.dna.affrc.go.jp/ap/mds) (Supplementary Figs. 13, 14, and 15). In the newer dataset, up-regulation of ethylene-related genes (e.g., *CmACO1*, *CmETR1/2*, and *CmNOR-NAC*) in ripe fruit was observed (Fig. 3b), consistent with ethylene production by the Harukei-3 fruit. Further co-expression analysis, including not only known ethylene-related genes but also 81 NAC domain, 90 homeobox, and 42 MADS-box transcription factors, identified a co-expression cluster that was specific to fruit ripening (Fig. 4a). In the central region of this cluster, we identified an AGAMOUS-like gene, *MELO3C019694.jh1*, which is a homolog of *Tomato AGAMOUS-LIKE 1* (*TAGL1*); a gene that has been shown to be involved in regulating fruit ripening^44,45^. Zhao et al.^21^ also recently identified this melon gene as a candidate QTL that regulates fruit suturing. A comparison of the Harukei-3 and DHL92 genomes indicated that Harukei-3 carries a longer protein-coding transcript of *MELO3C019694* relative to that of DHL92, and its expression was higher in ripe fruit than in pre-ripe fruit (Supplementary Fig. 16). When the genome sequences flanking *MELO3C019694* were analyzed using the Harukei-3 genome reference, the upstream promoter region of *MELO3C019694* was found to contain two Ty3-gypsy LTR-retroelements (Fig. 4b; chr11, 24,022,719–24,024,675 bp [1956 bp] and 24,024,698–24,031,639 bp [6941 bp]). Around this genomic region, two protein-coding sequences were identified. One of them, *MELO.jh102711.1*, encodes a protein sequence with retrotransposon-related protein domains such as IPR005162 (retrotransposon Gag domain), IPR013242 (retroviral aspartyl protease), and IPR000477 (reverse transcription). Together with LTR/gypsy elements, these sequences appear to function as LTR retrotransposons. According to the tissue-wide transcriptome dataset, *MELO3C019694* and the neighboring retrotransposon-related protein-coding sequences (e.g., *MELO.jh102711.1*) showed similar expression patterns, with the highest levels in post-harvest ripening fruits (Fig. 4c).

### PAPs and the expression of retrotransposon Gag sequences

**Fig. 5:**
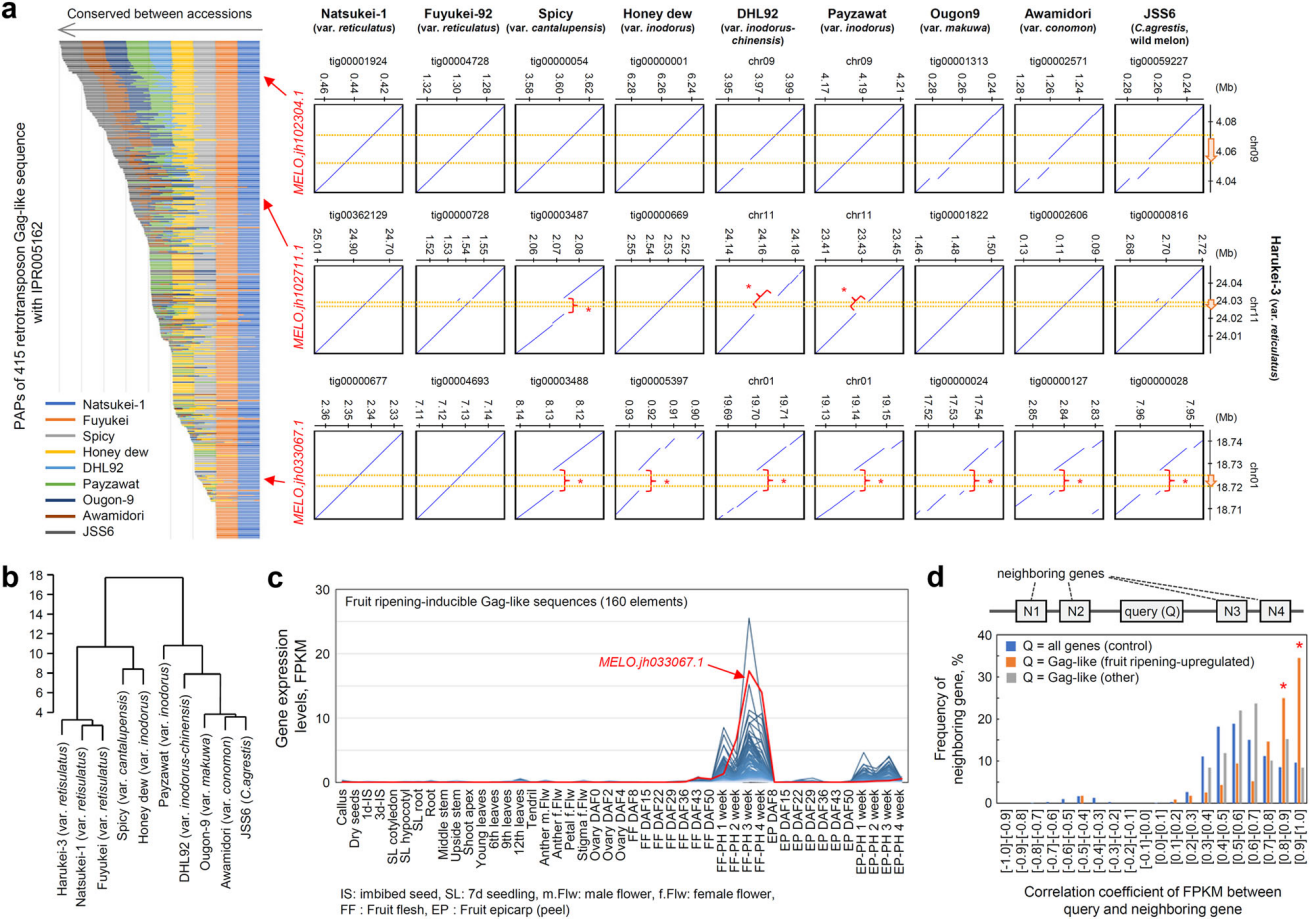
Presence/absence polymorphism (PAP) and fruit-ripening-inducible expression of retrotransposon Gag-like sequences. **a** Assembly-based PAP analysis of 415 retrotransposon Gag-like sequences between melon genomes. PAP was analyzed based on genomic DNA alignment using Harukei-3 as a reference. Except for the DHL92 and Payzawat genomes, genomic contigs were newly assembled based on the Oxford Nanopore technology sequencing data. The left graph shows the summary of the PAP analysis while the right panels show some examples of genomic alignments. In the right panels, genomic regions that are present in Harukei-3 but absent in other melons are indicated by red arrows and parentheses. In each alignment, the positions of Gag-like sequences are shown by yellow dashed lines. **b** A hierarchical clustering of 10 melon genomes based on the PAP genotyping dataset of Gag-like sequences. The PAP data of 415 Gag-like sequences shown in (**a**) were subjected to R-hclust analysis. **c** Fruit ripening-inducible expression of Gag-like sequences. Of the 415 Gag-like sequences found in the Harukei-3 genome, 160 show fruit-ripening-inducible expression. The expression of *MELO.jh033067.1* that was present in *reticulatus* melons but absent in other melons was strongly up-regulated in post-harvest ripening fruits (red arrow). **d** A histogram showing the correlation of gene expression between the Gag-like sequence and its neighboring genes. Correlation coefficients were calculated based on Fragments Per Kilobase of exon per Million mapped fragments (FPKM; gene expression levels) values from the tissue-wide transcriptome dataset shown in Fig. 3. Three different groups of queries were analyzed; all genes (control), fruit-ripening-inducible Gag-like sequences, and other Gag-like sequences (not up-regulated in ripening fruits, control). In the case of fruit-ripening-inducible Gag-like sequences, co-expression was observed for 59.4% of the neighboring genes (red asterisks).

The presence of the LTR retrotransposons in the upstream promoter sequence of *MELO3C019694.jh1* (*SlTAGL1* homolog) in the Harukei-3 genome prompted us to perform an enrichment analysis of CNV and PAP candidates (1,203 genes or putative protein-coding sequences) found between Harukei-3 and DHL92 genomes (Fig. 2a). For the purpose, a web-application tool, designated “GO term enrichment analysis”, was developed for the Melonet-DB database (https://melonet-db.dna.affrc.go.jp/ap/got). This revealed several InterPro IDs (IPRIDs) that are usually observed in retrotransposon-related function to be enriched in the 1203 candidates (e.g., IPR005162, IPR013242, and IPR000477; Supplementary Fig. 9b). This suggested that retrotransposons have been copied or jumped across chromosomes during diversification of the melon genome. Such structural difference should be detected in the form of PAPs between genomes if genome sequences are aligned and compared at a relatively narrow range (e.g., 50–100 kb). To analyze the PAPs of retrotransposon-related sequences in such a manner, we sequenced seven more melon genomes by using the ONT R9.4.1 platform. Genome assemblies were obtained as contig datasets for Natsukei-1 (var. *reticulatus*), Fuyukei (var. *reticulatus*), and Awamidori (var. *conomon*) in addition to Spicy, Honey dew, Ougon-9, Awamidori, and JSS6 (wild melon). The *N*_50_ values for the contig assemblies were more than 3.5 Mb, with a maximum value of 10.1 Mb (Supplementary Fig. 17), indicating that they were sufficient for local genomic sequence alignment analysis. According to the Harukei-3 ver. 1.41 genome reference, there are at least 415 putative protein-coding sequences with IPR005162 (retrotransposon Gag domain). Assembly-based sequence alignment analysis between Harukei-3 and other melons indicated that there are PAPs in these retrotransposon Gag-like sequences; some were conserved between melon accessions (e.g., *MELO.jh102304.1*) while others were not conserved and/or present in specific melons (e.g., *MELO.jh102711.1* and *MELO.jh033067.1*) (Fig. 5a). A hierarchical clustering analysis based on the PAP genotype dataset indicated that Natsukei-1 and Fuyukei melons are much closer to Harukei-3 compared to other accessions, which is consistent with the fact that they have the same origin (a *reticulatus* melon imported from the United Kingdom around 100 years ago) (Fig. 5b). By contrast, the Asian melons Awamidori, Ougon-9, and JSS6 are distant from such *reticulatus* melons. This result indicated that the PAP datasets obtained by assembly-based analysis reflect genomic variation between melon cultivars and accessions.

Then, tissue-wide gene expression patterns were analyzed in 415 retrotransposon Gag-like sequences. Interestingly, 160 (38.6%) showed fruit-ripening-inducible expression with the highest levels in post-harvest fruit samples (Fig. 5c). One of them, *MELO.jh033067.1*, was present only in *reticulatus* melons, but it showed higher expression levels in ripening fruits than did other genes (Fig. 5a, c). As described above, the expression pattern of *MELO.jh102711.1*, a Gag-like sequence, is similar to that of the neighboring gene, *MELO3C019694* (Fig. 4c). Thus, to investigate whether neighboring genes are co-expressed with Gag-like sequences, the correlation of gene expression was analyzed in a genome-wide manner. The result indicated that 59.4% of genes neighboring fruit-ripening-inducible Gag-like sequences also showed similar expression patterns (Fig. 5d and Supplementary Data 8; Pearson’s correlation coefficient *r* > 0.8). In contrast, in the case of other Gag-like sequences, the degrees of correlation between the Gag-like sequences and the neighboring genes were the same levels as the control.

### Heat-inducible expression of retrotransposon Gag sequences

**Fig. 6:**
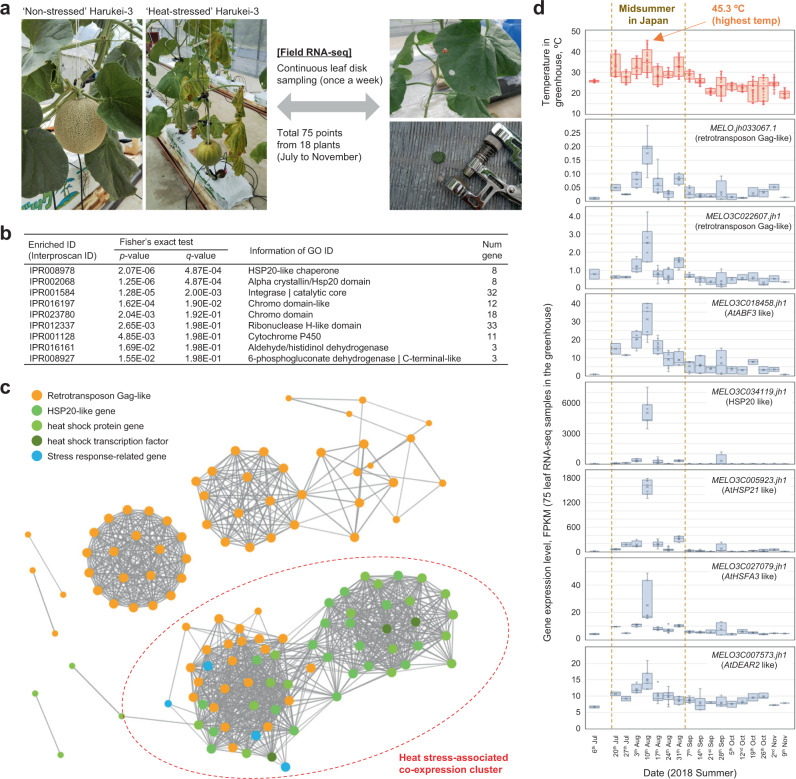
Transcriptional responses of retrotransposon Gag-like sequences in the greenhouse from July to November. **a** Harukei-3 melon plants under non-stressed or heat-stressed conditions. Leaf disks were collected weekly using a hole punch. In total, 75 RNA-seq data were obtained in 18 independent Harukei-3 plants from July to November. **b** InterPro ID enrichment analysis in the genes that co-express with retrotransposon Gag-like sequences. Co-expression dataset is based on 75 leaf RNA-seq data obtained in the greenhouse. Heat shock protein genes are highly enriched in the genes. **c** Co-expression of Gag-like sequences with abiotic stress-related genes. Gag-like sequences and abiotic stress-related genes are co-expressed in one cluster (shown by red dashed circle). **d** Changes in gene expression in the greenhouse. Expression patterns of two retrotransposon Gag-like sequences are shown together with those of abiotic stress-related genes such as *AtABF3*-like and *AtHSP21*-like.

There is increasing evidence that the expression of plant retrotransposon-related sequences is up-regulated by abiotic and biotic stress^46–48^. To investigate the environmental response of Harukei-3 retrotransposon-related sequences, we performed a field transcriptome analysis of leaf samples collected weekly from plants grown in a greenhouse at the University of Tsukuba from early summer to late autumn. In Japan, midsummer is not an appropriate season for melon cultivation because the temperature inside the greenhouse sometimes exceeds 45 °C. Indeed, Harukei-3 plants grown during midsummer showed severe signs of heat stress damage (Fig. 6a). In contrast, Harukei-3 plants grown during a cooler period (e.g., before midsummer or after September) had thick leaves with a dense green color, and produced sweeter melon fruit (Fig. 6a and Supplementary Fig. 1). We generated transcriptome data corresponding to a total 75 time points and 18 independent plants (Supplementary Data 9). WGCNA clustering using this dataset indicated that the retrotransposon Gag-like sequences were co-expressed with heat shock protein genes that carry the signature of HSP20-like chaperones (Fig. 6b, c). Homologs of *Arabidopsis thaliana* stress response regulators (e.g. *ABSCISIC ACID RESPONSIVE ELEMENT BINDING FACTOR 3* [*AtABF3*] and *DREB AND EAR MOTIF PROTEIN 2* [*AtDEAR2*]) were also co-expressed with these Gag-like sequences (Fig. 6c, d), indicating that melon plants grown during midsummer experienced drought stress, in addition to heat stress. Taken together, these results suggested that some retrotransposon Gag-like sequences were responsive to abiotic stress such as heat stress.

## Discussion

In this study, our assembly-based genome sequence comparison clearly demonstrated that there is a substantial PAPs in retrotransposon Gag-like sequences between melon genomes. For example, one of the Gag-like sequences, *MELO.jh33067.1*, is present in the genome of *reticulatus* melons (e.g., Harukei-3, Natsukei-1, and Fuyukei) but is absent from those of some melons, such as DHL92 and Payzawat (Fig. 5a). The genetic relationship was also well reflected in the hierarchical clustering dendrogram based on the PAP genotype dataset (Fig. 5b), demonstrating that our assembly-based approach is successful to analyze genome-wide PAPs between genomes. There is growing evidence that some plant LTR retrotransposons, which are classified into the subfamilies gypsy and copia, exhibit stress-inducible transcription^46–48^. The phytohormone ethylene has also been shown to be involved in stress-induced expression of retrotransposon in *Solanum chilense*^49^. If a retrotransposon is inserted into a promoter region, it may affect constituent *cis-*acting elements, which in turn may alter or enhance the transcriptional response of downstream genes to environmental and/or developmental signals^50^. Interestingly, most of the retrotransposon Gag-like sequences, including *MELO.jh33067.1*, showed the up-regulation of expression in ethylene-producing ripening fruits (Fig. 5c). Moreover, 59.4% of genes neighboring the fruit-ripening-inducible Gag-like sequences were co-expressed with the retrotransposon Gag-like sequences. Therefore, it is possible that the region of LTR retrotransposon around Gag-like sequences carry *cis*-acting and/or enhancer DNA elements that induce fruit-ripening-inducible gene expression. It seems also possible that ethylene production indirectly drives the transcription of Gag-like sequences. In plants, the biosynthesis of ethylene is also known to be stimulated under abiotic conditions, including heat stress^51^. Indeed, some of the Gag-like sequences were also up-regulated upon heat stress in melon (e.g., *MELO.jh33067.1* in Fig. 6d). In *A. thaliana*, heat-inducible transcription of the retrotransposon *ONSEN* is regulated through small interfering RNA^52,53^, and an epigenetic regulatory mechanism may similarly be involved in the regulation of LTR retrotransposon in melon. It is possible that such mechanism is a driving force to diversify the genome not only in melon but also in other plants.

The same also may be applicable to *MELO3C019694.jh1*, which is a homolog of tomato *AGAMOUS-*like gene *SlTAGL1*, a regulator of fruit ripening. Recently, Zhao et al.^21^ reported that *MELO3C019694.jh1* gene expression levels were higher in the sutured melon Vedrantais (var. *cantalupensis*) than in non-sutured Piel de Sapo (var. *inodorus*) at 7 days after pollination^21^. Here, we identified this gene as a possible regulator of fruit ripening as it is co-expressed with known ethylene-related fruit-ripening genes such as *CmACO1* and *CmNOR-NAC* (Fig. 4a). Since Vedrantais and Piel de Sapo are well-known climacteric and non-climacteric melons, respectively, it is possible that the difference in gene expression levels is associated not only with fruit suturing but also with ripening behavior (e.g., presence of ethylene production). Interestingly, the upstream promoter region of *MELO3C001864.jh1* in the Harukei-3 genome also carries at least one LTR retrotransposon insertion (e.g., *MELO.jh102711.1*). Given that this LTR retrotransposon is absent from the genomes of some melons such as DHL92 and Payzawat (Fig. 5a), it is possible that such PAP of the retrotransposon insertion is also involved in variation of gene expression levels between melon cultivars. Additional studies, using genome editing and/or comparative transcriptomics, will clarify the detailed role of this LTR retrotransposon in fruit ripening.

In this study, we present evidence that assembly level genome comparisons can elucidate structural genomic variation, including PAPs, as well as large genomic block duplications. ONT-based assembly successfully resolved structural variation (Figs. 1e, 2a, and 5a) and we show that ONT is also useful for genome-wide gene prediction, which is important for gene-based comparative genomics study (Fig. 2a). In particular, multiple ONT genome assemblies seem essential to analyze PAPs in a genome-wide manner (Fig. 5a). Information related to the Harukei-3 genome assembly, genome annotation, and the transcriptome dataset obtained in this study can be accessed using our updated web-application tools in the Melonet-DB database (https://melonet-db.dna.affrc.go.jp/). Together with future updates, this database will contribute to functional genomic study of melon, especially reverse genetics study using the genome editing technique and TILLING mutant population.

## Methods

### Plant materials and growth conditions

Seeds of cultivated melon accession, Harukei-3 (*C. melo* var. *reticulatus*), Natsukei-1 (var. *reticulatus*), Fuyukei (var. *reticulatus*), Honey dew (var. *inodorus*), Spicy (var. *cantalupensis*), Manshuu (var. *makuwa*), Ougon-9 (var. *makuwa*), Awamidori (var. *conomon*), and wild melon, JSS6 (*C. agrestis*), were obtained from the Genebank of the National Agriculture and Food Research Organization (NARO) in Japan. Melon plants were grown using the hydroponics method in the greenhouse of the University of Tsukuba in Japan as previously reported^18^. For genomic DNA sequencing, apexes of branched shoots were detached from plants and immediately frozen in liquid nitrogen. For tissue-wide RNA-seq study, tissues shown in Fig. 3a and Supplementary Fig. 6 were similarly obtained and frozen in liquid nitrogen. For field RNA-seq study, hole-punched leaf samples were also collected in a weekly manner from July to November as shown in Fig. 6. These samples were crushed to powdery frozen samples using the Multi-beads shocker instrument (Yasui Kikai Corporation, Osaka, Japan) and stored at −80 °C until use.

### Genomic DNA isolation and DNA sequencing

Genomic DNA was isolated using Maxwell^®^ 16 Tissue DNA Purification Kit (Code No. AS1030, Promega, Wisconsin, USA). Although this kit is designed to couple with an automated DNA extraction machine, we did not use this but manually isolated genomic DNA by hand to obtain long intact DNA. Isolated genomic DNA was further subjected to the Short read Eliminator XS kit (Circulomics, Maryland, USA) to remove short DNA fragments with <5 kb. For ONT sequencing, the DNA library was prepared using the Ligation sequencing kit (Code No. SQK-LSK109, ONT, Oxford, UK) according to the manufacturer’s protocol. DNA sequencing run was performed with a Nanopore Minion^®^ device coupled with flow cell. For the genome sequencing of Harukei-3, both R9.4.1 and R10 flow cells were used, whereas only R9.4.1 was used in the sequencing of Natsukei-1, Fuyukei, Spicy, Honey dew, Awamidori, and JSS6. ONT flow cells were repetitively used at least twice with the same DNA library. To obtain DNA sequence data, basecalling was performed using a CUDA-enabled GPU server with ONT’s guppy ver. 3.3.0 software. For PacBio RSII and Illumina paired end (PE) sequencing, the outsourcing service of Macrogen Japan Co. Ltd (Kyoto, Japan). was used except the Illumina PE data of Harukei-3 that was obtained with a Hiseq-2000 sequencer at Cornel University. Illumina sequencing was performed with the 100 bp PE mode in Harukei-3 or the 150 bp PE mode in Honey dew, Spicy, Manshuu, Ougon-9, and JSS6. Illumina mate pair sequencing was performed with 5 kb and 10 kb insert libraries by using the outsourcing service of Hokkaido System Science Co. Ltd. (Sapporo, Japan).

### RNA isolation and RNA-seq data acquisition

Total RNA was isolated using Maxwell^®^ 16 LEV Plant RNA Kit (Code No. AS1430, Promega, Wisconsin, USA) according to the manufacturer’s protocol. For ONT direct RNA-seq and cDNA RNA-seq, libraries were prepared using the Direct RNA sequencing kit (Code No. SQK-RNA002, ONT, Oxford, UK) or the Direct cDNA sequencing kit (Code No. SQK-DCS108), respectively, according to the manufacturer’s protocol. To obtain RNA sequence data, basecalling was performed with ONT’s Guppy ver. 3.2.1 software. For Illumina RNA-seq with the 150 bp PE mode, the outsourcing service of Macrogen Japan Co. Ltd. was used.

### Whole genome assembly

The procedures, datasets, and detailed parameters used for whole genome assembly of Harukei-3 are summarized in Supplementary Fig. 2. We used two kinds of sequence reads, ONT (R9.4.1 + R10 flow cells; 32.6 Gb) and PacBio RSII (19.5 Gb), for initial contig assembly. In the case of ONT, reads with ≥5 kb were selected and used. Contigs were separately assembled based on ONT or PacBio reads with the Canu ver. 1.8 pipeline^54^; then errors present in the contig sequences were corrected with Pion^55^ using 37 Gb of Illumina PE dataset. At this point, contig *N*_50_ values for ONT-based and PacBio-based contig assemblies were 8.6 Mb and 0.86 Mb, respectively. Then, scaffolds were assembled based on contigs by combining methods of Bionano Irys optical map and Illumina mate pair (Supplementary Fig. 2). For Bionano scaffolding, 86 Gb raw data were obtained using the outsourcing service of AS ONE Corp. (Osaka, Japan). They were first assembled to construct “cmap” with Irys solve ver. 3.2; then, cmap was used for both scaffolding and correction of chimeric contigs (incorrectly assembled contigs) using the same software. Scaffolding was also performed using 74 Gb of Illumina mate pair (5 kb and 10 kb insert sizes) with SSPACE ver. 3.0^56^ at different “*k*” parameter values (from *k* = 160 to 80, 40, 20, 10, and 5). By using *k* values from larger to smaller in series, we tried to maximize the connections and minimize false positives. Chimeric scaffolds generated by the mate pair scaffolding were again corrected using Bionano cmap. At this point, scaffold *N*_50_ values for ONT-based and PacBio-based assemblies were 17.5 Mb and 11.4 Mb, respectively. ONT-based scaffolds were further updated using PacBio-based scaffolds as a hint. In this attempt, both scaffolds were first classified into each chromosome group by using linkage map information. Then, candidates of PacBio-based scaffolds that can connect two distinct ONT-based scaffolds were identified by BLAST-n search using the following conditions: *p*-value ≤ 1e–150, sequence identity ≥ 99%, blast score ≥ 1000, and alignment length ≥ 5000 bp (Supplementary Fig. 2). If both end of the PacBio-based scaffold had sequence alignments with distinct ONT-based scaffolds, it was used to connect them. Finally, the chromosome-scale pseudomolecule was constructed using 28 genomic scaffolds that were anchored and oriented by linkage map information^16,30,57^.

For the contig assembly in Natsukei-1, Fuyukei, Spicy, Honey dew, Ougon-9, Awamidori, and JSS6, ONT sequencing reads with ≥5 kb (R9.4.1 flow cell) were first subjected to the Canu ver. 1.8 pipeline. The resultant contig sequences were subjected to Racon^58^ and Medaka (https://nanoporetech.github.io/medaka/) to correct erroneous bases. To further determine the candidates of chimeric contigs, ONT reads were aligned to contig sequences with minmap2^59^ using the following parameter: “-a -uf -k14 -A 2 -B 4 -O 4,24 -E 2,1”. Then, read depth data were obtained based on the read alignment information using the mpileup function of samtools^60^. Contigs were split at positions where the depth of ONT reads was less than four.

### Gene prediction

Procedures and datasets used for genome annotation are summarized in Supplementary Figs. 6, 7. For ONT-based gene prediction, datasets of 8.2 Gb ONT direct RNA-seq and 8.8 Gb ONT cDNA RNA-seq were combined and used. They were aligned to Harukei-3 ver. 1.41 genomic sequence with Minimap2 using the following parameters “-ax splice -uf -k14.” Then, transcript information with exon-intron structure was obtained by pinfish (https://github.com/nanoporetech/ont_tutorial_pinfish) using several “*c*” parameter values (*c* = 2, 3, 5, and 10; Supplementary Fig. 7). Predicted transcript sequences were obtained from the genomic sequence with gffread (https://ccb.jhu.edu/software/stringtie/gff.shtml#gffread) based on the General Feature Format (GFF) information of Pinfish; then, they were combined with transcript sequence information of DHL92 genome annotation CM4.0. Again, Minimap2 alignment and Pinfish prediction were performed based on the combined transcript sequences to obtain the merged GFF annotation. Next, the protein-coding open reading frame (ORF) was predicted in each transcript followed by hmmsearch. The best possible ORFs were kept by selecting those with the highest sum total hmmsearch scores. If no protein domain was found in any ORF candidates, the longest ORF was kept. Transcripts were further grouped into gene units based on the position of the exon(s) and the results of the self-BLAST search (both transcript and protein sequences); transcripts were grouped if both the transcript and protein sequences had homology to each other and the positions of exon(s) on the genome were consistent between them. To further select the best-possible ORF in each gene unit, hmmsearch as well as BLAST-p search against protein sequences of 9 plant genomes were performed again (a list of the genome references used for the purpose is shown in Supplementary Fig. 7d). The ONT-based method described above predicted 31,306 protein-coding genes. A perl pipeline designated “ONT4genepredict” was developed to automatically perform the information analysis described above. It is available in Melonet-DB (https://melonet-db.dna.affrc.go.jp/ap/dnl). To evaluate the completeness of predicted genes, we used BUSCO ver. 3.0 benchmark^32^. The protein BUSCO score for the ONT-based gene dataset was 1362 (94.6%) (Supplementary Fig. 7c). In addition to the ONT-based method, gene prediction was also performed with AUGUSTUS ver. 3.3.2 (ab initio method)^61^, Braker2 pipeline^62^, and Genome Threader^63^. For AUGUSTUS gene prediction, the parameter dataset was first trained and generated with the perl script autoAug.pl using the ONT-based annotation dataset described above. Then, genes were predicted with AUGUSTUS software using the default parameters. Braker2 was executed using the Illumina RNA-seq dataset of 45 tissue-wide samples (total 118 Gb; Fig. 3a). RNA-seq reads were first aligned to the Harukei-3 genome sequence, then the read alignment information was merged and used for Braker2 gene prediction. Transcript annotation was also obtained with StringTie^64^ software based on the read alignment information. Genome Threader was executed based on the protein sequences of 10 published plant genomes (listed in Supplementary Fig. 7d). Then, EvidenceModeler (EVM, https://evidencemodeler.github.io/) was used to integrate the results of StringTie, AUGUSTUS, Braker2, and Genome Threader with the weight score setting of 10, 8, 1, and 1. EVM produced the dataset of 59,613 protein-coding genes with complete BUSCO ver. 3.0 score = 1348 (93.6%). Finally, using the EVM-based dataset as a supplementary dataset, ONT-based annotation dataset was updated to obtain 33,829 protein-coding genes (40,363 transcripts, BUSCO ver. 3.0 score = 1,372 [95.3%]). InterProScan^33^ was also conducted to obtain GO and InterPro ID in each predicted protein amino acid sequence.

### Identification of repetitive elements

Repetitive elements including DNA/RNA transposable elements were searched in both Harukei-3 ver. 1.41 and DHL92 CM3.6.1 genomes using RepeatModeler and RepeatMasker (http://www.repeatmasker.org/) using a repeat sequence dataset, dc20181026.

### RNA-seq and co-expression data analysis

Alignment of Illumina RNA-seq paired end short reads was performed with HISAT2^65^ using the following parameters: “–maxins 1000 - –score-min L,0,−0.12 –mp 2,2 –np 1 –rdg 1,1 –rfg 1,1.” Then, gene expression levels were calculated as FPKM values with StringTie. After removing non-expressing genes (e.g., FPKM < 0.1 in any of the samples), WGCNA was performed to obtain the co-expression dataset as described previously^18^. Pearson’s correlation coefficients were also calculated using R 3.2.3 (https://www.r-project.org/) based on FPKM values independently of WGCNA to distinguish positive and negative correlations. Co-expression datasets can be explored using the web-application tool “Co-expression viewer” in the Melonet-DB (https://melonet-db.dna.affrc.go.jp/ap/mds).

### Resequencing

Three different melon genome references were used in this study: Harukei-3, DHL92 (CM3.6.1 genome sequence + CM4.0 annotation), and Payzawat (ASM976082v1 genome sequence). Illumina paired end short reads of Harukei-3, Honey dew, Spicy, Manshuu, Ougon-9, and JSS6 (see above) were aligned to genome sequence with bowtie2^66^ using the following parameters, “–end-to-end –very-sensitive –score-min L,0,−0.12 –mp 2,2 –np 1 –rdg 1,1 –rfg 1,1.” Variant call was performed with the Genome analysis tool kit^67^, and mutation characterization was performed as described previously^68^.

### Comparative genomics analysis

Genomic alignment was performed with LAST^69^ using the following parameters, “-e 25 -v -q 3 -j 4 -P 32 -a 1 -b 1 (for lastal)” and “-s 35 -v (for last-split)”. After file format conversion via maf-convert, plot graphs comparing distinct genomes were generated with R 3.2.3 based on the result of LAST alignment. To obtain information of orthologue partners, we performed bidirectional blast searches based on both transcript and protein sequences. Because it is difficult to determine one-to-one orthologue partners when genes are duplicated in either genome, we also used the information of transcript alignment obtained with blat^42^ as supporting information. When genes were paired in both bidirectional BLAST-n/p search and transcript alignment analysis, they were determined as one-to-one orthologue partners. Candidates of CNV and PAP were determined by integrating the results of bidirectional BLAST-n/p search with the information of gene position on the chromosome that could be obtained from genome annotation (GFF3 files). Information analysis described above has been automated with Perl scripts. Circos^70^ was used to visualize and compare genomes.

For PAP analysis of retrotransposon Gag-like sequences, DNA alignment analysis was performed for each sequence in nine melon genomes using the Harukei-3 genome as a standard reference. First, the genomic sequence containing the Gag-like sequence and its surrounding region (approx. 50–100 kb) was obtained from the Harukei-3 genome sequence. By using this sequence as a query, a BLAST-n search was performed against the whole genome sequence data of the target melon cultivar or accession. The specific region of contig or chromosome that showed the best homology to the query sequence was identified from the BLAST search result, then the DNA sequence of this region was obtained and further used for LAST alignment. Plot graphs comparing both sequences (Harukei-3 versus the target melon) were generated with R 3.2.3 based on the result of the LAST alignment. The PAP status of the Gag-like sequence in the target melon was also calculated as a numeric value based on the alignment ratio of the corresponding genomic region. For example, an alignment ratio of 1.0 means the complete presence of the Gag-like sequence in the target melon genome, while 0 means that the sequence is absent.

### InterPro ID enrichment analysis

ID enrichment analysis was performed based on Fisher’s exact test using the R-exact2x2 module. To calculate *q*-value from *p*-value, R-qvalue package was used. This ID enrichment analysis is available in the “GO enrichment analysis tool” in Melonet-DB (https://melonet-db.dna.affrc.go.jp/ap/got).

### Statistics and reproducibility

All statistical tests were performed using available softwares, packages, and online tools mentioned in the methods. Reproducibility can be accomplished using raw sequencing data deposited on public databases and the same command lines mentioned in the methods, where we used publicly available softwares for most of the analysis. Fisher’s exact test was used for testing enriched GO or InterPro terms. Both *p*-value and *q*-value was used to indicate statistical significance. The number of RNA-seq samples used for tissue-wide or leaf co-expression analyses were 45 and 75, respectively, which were determined according to the previous study^18^. Co-expression was evaluated based on the weight values calculated by R-WGCNA and pearson’s correlation coefficients (*n* = 45 or 75).

### Reporting summary

Further information on research design is available in the Nature Research Reporting Summary linked to this article.

## Supplementary information

Supplementary Information

Supplementary Data 1

Supplementary Data 2

Supplementary Data 3

Supplementary Data 4

Supplementary Data 5

Supplementary Data 6

Supplementary Data 7

Supplementary Data 8

Supplementary Data 9

Reporting Summary

## Data Availability

Raw sequencing data used in this study can be found in the NCBI database under the following Bioproject accession numbers: PRJNA603155 (genome sequencing dataset of Harukei-3 melon), PRJNA624817 (genome sequencing dataset of seven melon accessions), PRJNA603146 (ONT cDNA RNA-seq), PRJNA603129 (ONT direct RNA-seq), PRJNA603204 (tissue-wide RNA-seq of Harukei-3 melon), or PRJNA603202 (leaf RNA-seq in the greenhouse). Genome assembly and annotation of Harukei-3 melon (ver. 1.41 genome reference) is available on Melonet-DB (https://melonet-db.dna.affrc.go.jp/ap/dnl).

## Code availability

Web-application tools used in this study are available on Melonet-DB (https://melonet-db.dna.affrc.go.jp) or its mirror site (https://gene.melonet-db.jp/). The ONT4genepredict pipeline that can be used to predict genes based on ONT RNA-seq is also available on this web site.
