# Supplementary material for: Comparative genomics of muskmelon reveals a potential role for retrotransposons in the modification of gene expression
Source: Commun Biol. 2020 Aug 13;3:432. doi: 10.1038/s42003-020-01172-0 (PMC7426833; doi:10.1038/s42003-020-01172-0)
Supplement: Supplementary file 1 — Supplementary Information [file 42003_2020_1172_MOESM1_ESM.pdf]

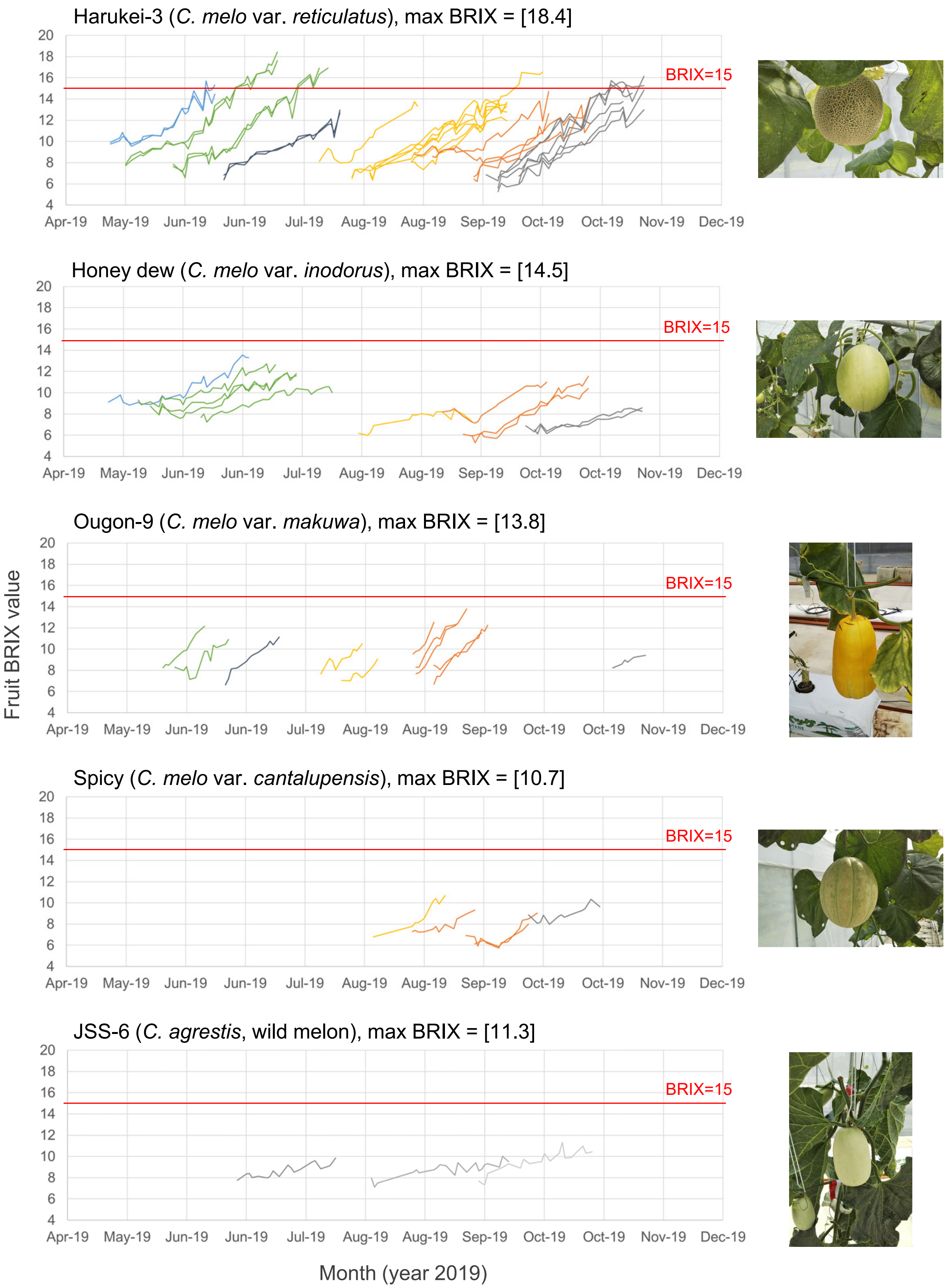

Supplementary Fig. 1 Seasonal changes in fruit BRIX

Seasonal changes in fruit BRIX in five melon accessions. Fruit BRIX was continuously analyzed in a non-destructive manner using ‘Fruit selector’ near-infrared spectrometer (Kubota Co. Ltd., Japan) from April to November in 2019 in the greenhouse at the University of Tsukuba. Line colors distinguish the timing of fruit set from Spring to late Autumn. The melon accessions analyzed were Harukei-3, Honey dew, Ougon-9, Spicy, and JSS-6.

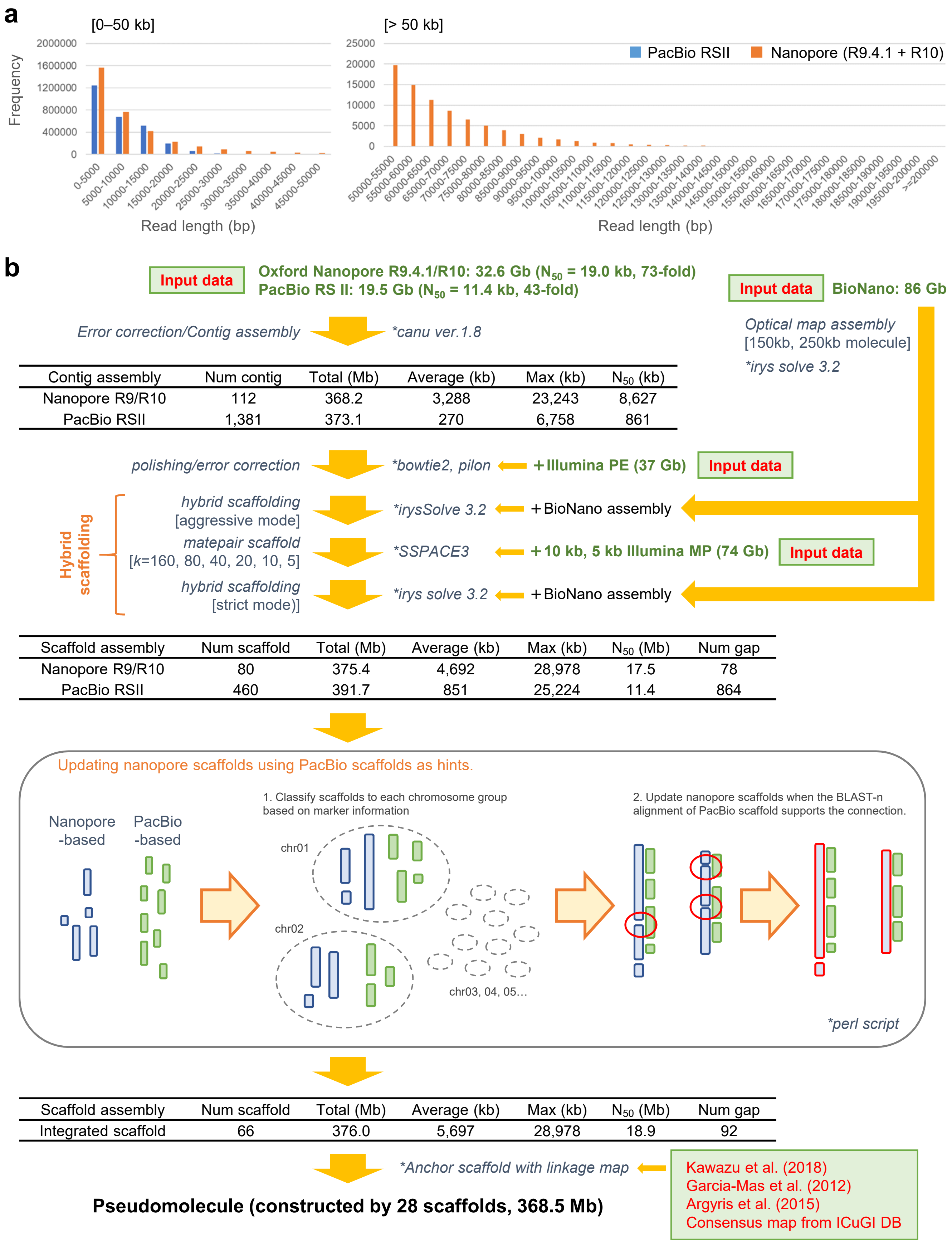
a
[0–50 kb]
[> 50 kb]
Frequency
Read length (bp)
PacBio RSII
Nanopore (R9.4.1 + R10)
b
Input data
Oxford Nanopore R9.4.1/R10: 32.6 Gb (N_{50} = 19.0 kb, 73-fold)
PacBio RS II: 19.5 Gb (N_{50} = 11.4 kb, 43-fold)
Input data
BioNano: 86 Gb
Error correction/Contig assembly
*canu ver.1.8
Optical map assembly
[150kb, 250kb molecule]
*irys solve 3.2
Contig assembly | Num contig | Total (Mb) | Average (kb) | Max (kb) | N_{50} (kb)
Nanopore R9/R10 | 112 | 368.2 | 3,288 | 23,243 | 8,627
PacBio RSII | 1,381 | 373.1 | 270 | 6,758 | 861
polishing/error correction
*bowtie2, pilon
+Illumina PE (37 Gb)
Input data
Hybrid scaffolding
hybrid scaffolding
[aggressive mode]
*irysSolve 3.2
+BioNano assembly
matepair scaffold
[k=160, 80, 40, 20, 10, 5]
*SSPACE3
+10 kb, 5 kb Illumina MP (74 Gb)
Input data
hybrid scaffolding
[strict mode)]
*irys solve 3.2
+BioNano assembly
Scaffold assembly | Num scaffold | Total (Mb) | Average (kb) | Max (kb) | N_{50} (Mb) | Num gap
Nanopore R9/R10 | 80 | 375.4 | 4,692 | 28,978 | 17.5 | 78
PacBio RSII | 460 | 391.7 | 851 | 25,224 | 11.4 | 864
Updating nanopore scaffolds using PacBio scaffolds as hints.
Nanopore -based
PacBio -based
1. Classify scaffolds to each chromosome group based on marker information
chr01
chr02
chr03, 04, 05…
2. Update nanopore scaffolds when the BLAST-n alignment of PacBio scaffold supports the connection.
*perl script
Scaffold assembly | Num scaffold | Total (Mb) | Average (kb) | Max (kb) | N_{50} (Mb) | Num gap
Integrated scaffold | 66 | 376.0 | 5,697 | 28,978 | 18.9 | 92
*Anchor scaffold with linkage map
Kawazu et al. (2018)
Garcia-Mas et al. (2012)
Argyris et al. (2015)
Consensus map from ICuGI DB
Pseudomolecule (constructed by 28 scaffolds, 368.5 Mb)

Supplementary Fig. 2 Procedure of whole genome assembly in Harukei-3

a Histograms of sequenced reads in Oxford Nanopore technology (ONT, R9.4.1 and R10 flow cells) and PacBio RSII. Reads with > 50 kb are present only in ONT reads (right). **b** Procedure of genome assembly. Contigs were separately assembled based on ONT or PacBio reads; then, scaffolds were assembled using BioNano optical map and Illumina mate-pair. ONT-based scaffolds were further updated by using Pacbio-based scaffolds as a hint; both scaffolds were first classified into each chromosome group based on the linkage marker information[1,2,3], then hint scaffolds (Pacbio-based) that connect two different ONT-based scaffolds were identified based on BLAST-n alignment. Finally, the chromosome-scale pseudomolecule was constructed by 28 genomic scaffolds that were anchored and oriented using the linkage map information[1,2,3].

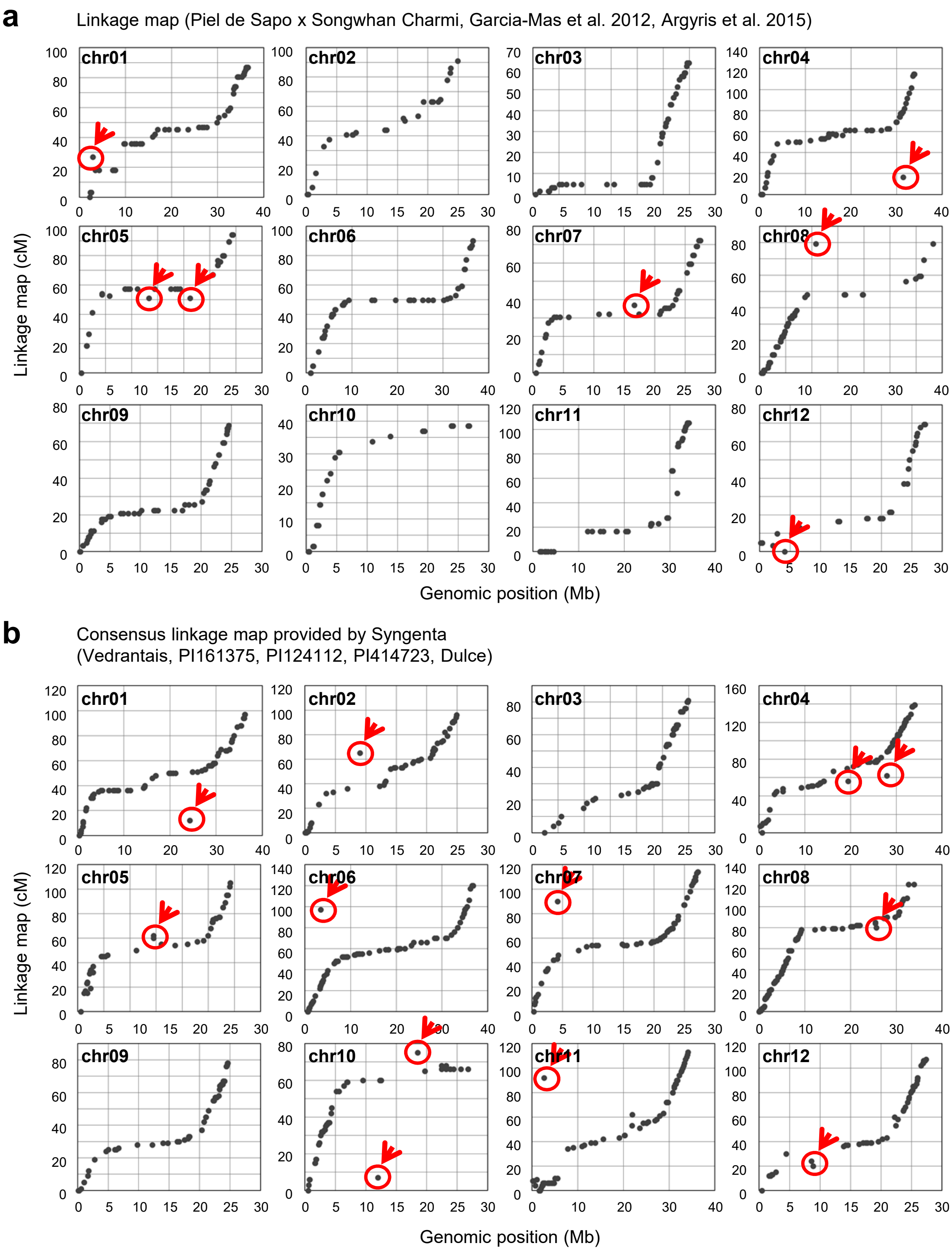

Supplementary Fig. 3 Comparison of Harukei-3 genome sequence with linkage map information

a, b Comparison of Harukei-3 genome sequence with linkage maps. Linkage maps were derived from a cross between Piel de Sapo and Songwhan Charmi[3] (a) or the consensus genetic map available in Cucurbit Genomics Database[4,5] (b). Red circles and arrows indicate genetic markers in which the physical position does not match the linkage map position.

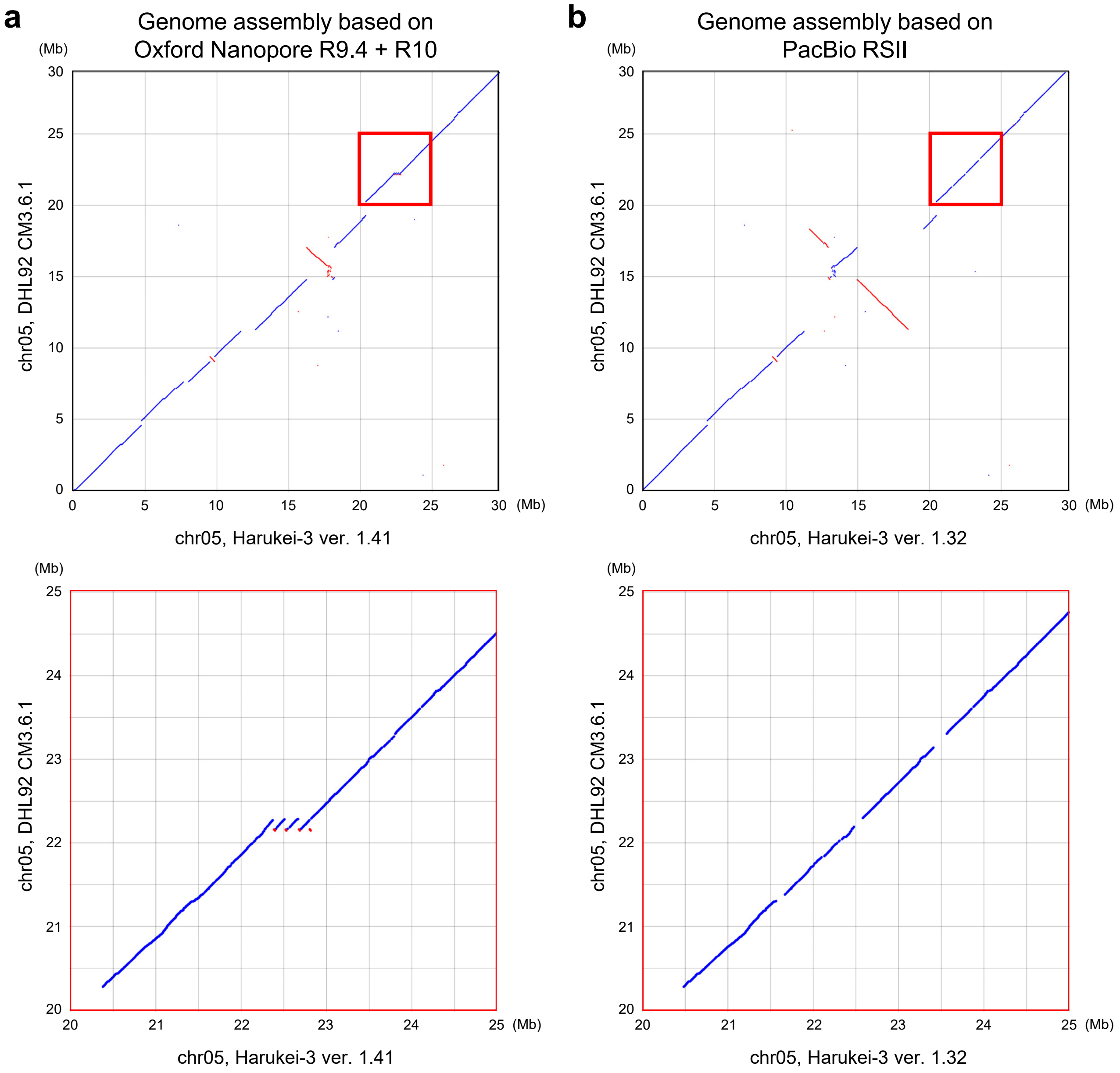

Supplementary Fig. 4 Comparison of genome assembly between ONT and PacBio in Harukei-3

a, b Genomic alignments of chromosome 5 between DHL92 and two versions of Harukei-3. The Harukei-3 sequences are ONT-based (a, ver. 1.41) or PacBio RSII-based (b, ver. 1.32). Entire chromosome (top) and the magnified view of the specific region (20–25 Mb) (bottom) are shown. ONT-based Harukei-3 assembly can resolve large genome block duplications of around 22–23 Mb, whereas it is absent in the PacBio-based assembly. Blue and red dots indicate that DNA is aligned in the forward or reverse directions, respectively.

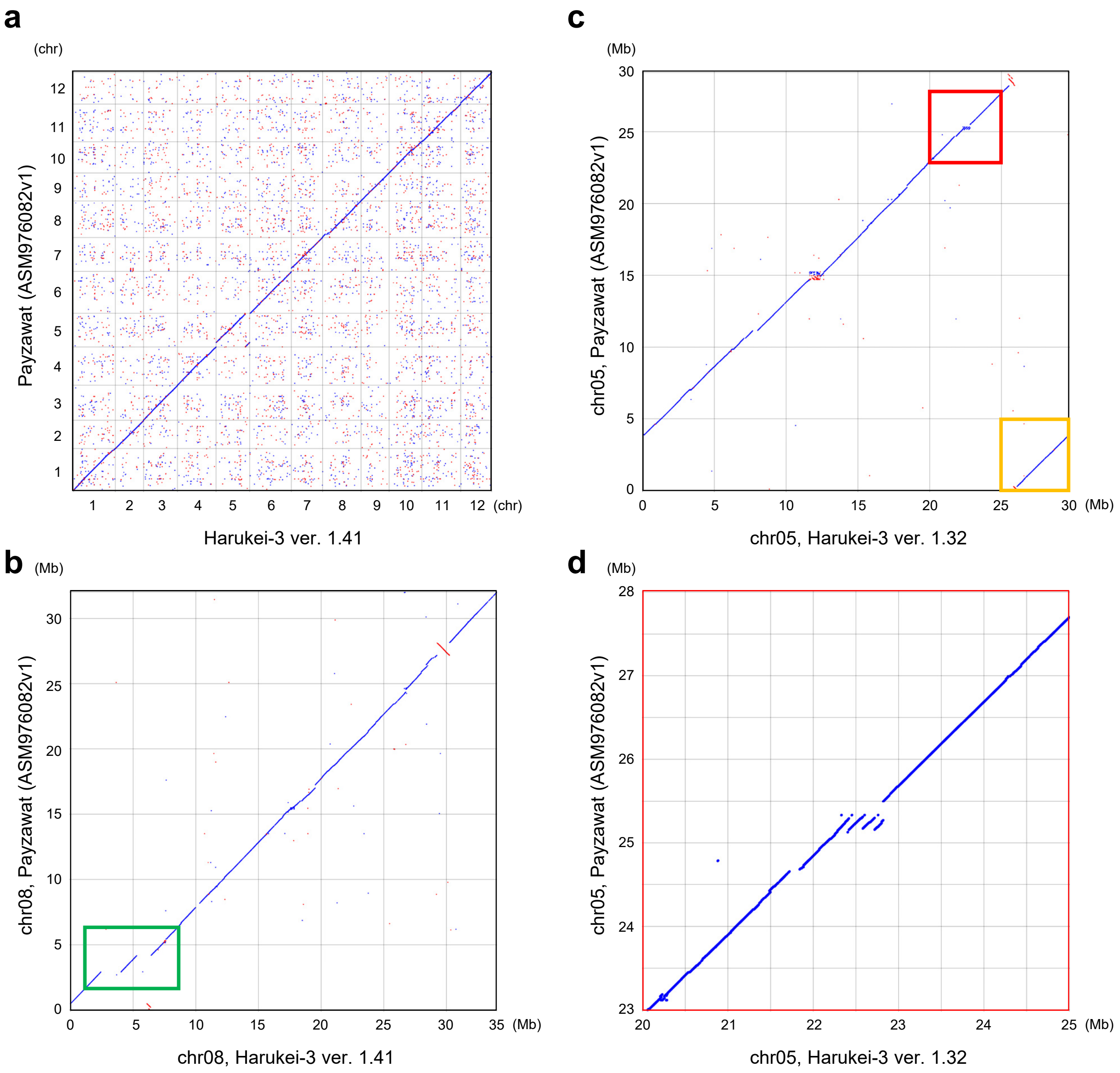

Supplementary Fig. 5 Genomic alignment between Harukei-3 and Payzawat melon genomes

a, b, c Genomic alignment of entire chromosomes (a), chromosome 8 (b), or chromosome 5 (c) between Harukei-3 (ver. 1.41) and Payzawat[6] (ASM976082v1) genomes. Large genomic blocks that are present in Harukei-3 but not in Payzawat genome are indicated by the green rectangle in b. Inter-chromosomal translocation observed in chromosome 5 between the two genomes is indicated by the yellow rectangle in c. **d** Magnified view of the specific region of chromosome 5 (20–25 Mb in in Harukei-3, 23–28 Mb in Payzawat; shown by the red rectangle in c. Harukei-3 genome can resolve large genomic block duplications around 22–23 Mb, but it is absent or not assembled in the Payzawat genome. Blue and red dots indicate that DNA is aligned in the forward or reverse directions, respectively.

a

Combined RNA set1

Sample	Condition	Bulked replicate	Total RNA (µg)
Seedling cotyledon	7d after imbibition on filter paper	2 x 4 seedlings	20
Seedling root	7d after imbibition on filter paper	2 x 4 seedlings	20
Seedling hypocotyl	7d after imbibition on filter paper	2 x 4 seedlings	20
Germinating seed	1d imbibition on filter paper	2 x 4 seedlings	30
Germinating seed	3d imbibition on filter paper	1 x 4 seedlings	30
Dry seed	-	1 x >100 seeds	20

Combined RNA set2

Sample	Condition	Bulked replicate	Total RNA (µg)
Expanded leaves (6th)	Hydroponically-cultivated in the greenhouse	3	15
Expanded leaves (9th)	Hydroponically-cultivated in the greenhouse	3	15
Expanded leaves (12th)	Hydroponically-cultivated in the greenhouse	3	15
Hole-punched leaves	infected with powdery mildew	8	15
Hole-punched leaves	without powdery mildew	8	15
Root	Hydroponically-cultivated in the greenhouse	4	25
Main stem	Hydroponically-cultivated in the greenhouse	2	16
Tendril	Hydroponically-cultivated in the greenhouse	2	6

Combined RNA set3

Sample	Condition	Bulked replicate	Total RNA (µg)
Anther (female)	just after flowering	4 x >10 flowers	12.5
Anther (male)	just after flowering	4 x >10 flowers	12.5
Ovary (female)	just after flowering	3 x 4 ovaries	25
Petal	just after flowering	4 x >10 flowers	25
Pistil (female)	just after flowering	3 x 4 ovaries	25

Combined RNA set4

Sample	Condition	Bulked replicate	Total RNA (µg)
Fruit peel	DAF8	2	4
Fruit peel	DAF15	2	4
Fruit peel	DAF22	2	4
Fruit peel	DAF29	2	2.5
Fruit peel	DAF36	2	2.5
Fruit peel	DAF43	2	2.5
Fruit peel	DAF50	2	2.5
Fruit peel	Post harvest, 3weeks	2	2.5
Fruit peel	Post harvest, 1week	2	2.5
Fruit peel	Post harvest, 4weeks	2	2.5
Fruit peel	Post harvest, 2weeks	2	2.5
Fruit	DAF4	4 ovaries	10
Fruit flesh	DAF8	2	4
Fruit flesh	DAF15	2	4
Fruit flesh	DAF22	2	4
Fruit flesh	DAF29	2	2.5
Fruit flesh	DAF36	2	2.5
Fruit flesh	DAF43	2	2.5
Fruit flesh	DAF50	2	2.5
Fruit flesh	Post harvest, 3weeks	2	2.5
Fruit flesh	Post harvest, 1week	2	2.5
Fruit flesh	Post harvest, 4weeks	2	2.5
Fruit flesh	Post harvest, 2weeks	2	2.5

Combined RNA set5

Sample	Condition	Bulked replicate	Total RNA (µg)
Hole-punched leaves	infected with powdery mildew (2018-Apr)	4	50
Hole-punched leaves	infected with powdery mildew (2018-Oct)	4	50

Combined RNA set6

Sample	Condition	Bulked replicate	Total RNA (µg)
Etiolated seedling	germinted in the dark	>10 seedlings	100

Combined RNA set7

Sample	Condition	Bulked replicate	Total RNA (µg)
Shoot apex	Hydroponically-cultivated in the greenhouse	4	130

ONT direct RNA-seq (R9.4.1 flow cell)

1. Combine RNA in each set
2. Poly-A+ purification
3. Nanopore RNA-seq library prep.
4. Sequencing
5. Basecall with guppy

Total 8.2 Gb, 7.6 million reads
(five flow cells)

b

RNA (hole-punshed leaves)

Sample	Condition	Bulked replicate	Total RNA (µg)
Harukei-3 (rep.1)	Hydroponically-cultivated in the greenhouse	4	>100
Harukei-3 (rep.2)	Hydroponically-cultivated in the greenhouse	4	>100
Harukei-3 (rep.3)	Hydroponically-cultivated in the greenhouse	4	>100
Harukei-3 (rep.4)	Hydroponically-cultivated in the greenhouse	4	>100
Honey dew (rep.1)	Hydroponically-cultivated in the greenhouse	4	>100
Honey dew (rep.2)	Hydroponically-cultivated in the greenhouse	4	>100
Awamidori (rep.1)	Hydroponically-cultivated in the greenhouse	4	>100
Awamidori (rep.2)	Hydroponically-cultivated in the greenhouse	4	>100
Ougon-9 (rep.1)	Hydroponically-cultivated in the greenhouse	4	>100
Ougon-9 (rep.2)	Hydroponically-cultivated in the greenhouse	4	>100
JSS-6 (rep.1)	Hydroponically-cultivated in the greenhouse	4	>100
JSS-6 (rep.1)	Hydroponically-cultivated in the greenhouse	4	>100

ONT cDNA-seq (R9.4.1 flow cell)

1. Poly-A+ purification
2. Nanopore RNA-seq library prep.
3. Multiplex sequencing
4. Basecall with guppy

Total 8.8 Gb, 11.5 million reads
(one flow cell)

Supplementary Fig. 6 Summary of RNA samples used for ONT RNA-seq in Harukei-3
a, b Summary of RNA samples used for ONT direct RNA-seq (a) or cDNA RNA-seq (b). Both RNA-seq datasets were used for gene prediction in the Harukei-3 genome. Sequencing strategies are also briefly described on the left.

a

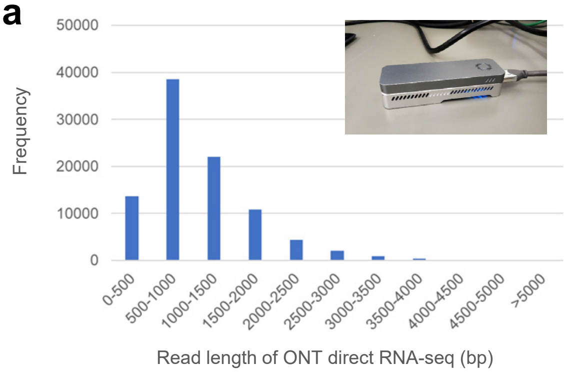

b

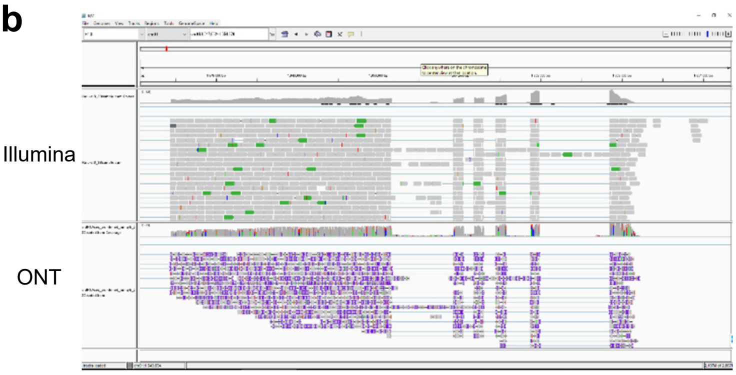

c

Gene prediction based on ONT RNA-seq

Oxford Nanopore® direct RNA-seq
Eight runs with 5 flow cells (R9.4.1)
Total 8.2 Gb, 7.6 million reads

↓

Alignment to Harukei-3 ver. 1.41 genome
(Minimap2 with parameters '-ax splice -uf -k14')

↓

Prediction of mRNA (exon-intron) structure based on ONT read alignment
(pinfish with different parameters '*c* = 2,3,5,10')

↓

Integrate pinfish results between '*c* = 2,3,5,10' and protein coding DNA sequences from DHL92 CM4.0

↓

ORF search followed by hmmsearch to keep the best possible ORF with known protein domain
(Longest ORF is kept in case of no protein domain)

↓

Transcripts are grouped into gene unit based on BLAST-n/p search and genomic position information

↓

Best-possible ORF are selected again based on hmmsearch and BLAST-p search against protein sequences from 9 plant genomes

↓

[i] 31,306 protein-coding genes (36,826 transcripts)
1,437 non-coding genes

Gene prediction using other methods

AUGUSTUS
Parameter training was first performed with ONT-based annotation obtained in [i] then genes were predicted with AUGUSTUS ver. 3.3.2.

Braker2
Reads of tissue-wide Illumina Hiseq® RNA-seq (45 samples, total 118 Gb, 920 million reads) were first aligned to Harukei-3 ver. 1.41 sequence with hisat2, then genes were predicted with braker2.

Genome threader
Protein sequences of 10 plant genomes were aligned to Harukei-3 ver. 1.41 genome sequence.

StringTie
Transcript annotation was obtained with StringTie based on the HISAT2 read alignment of tissue-wide Illumina Hiseq® RNA-seq (45 samples).

Integrate annotation datasets with Evidence modeler using the following parameter condition

TRANSCRIPT	StringTie	10
ABINITIO_PREDICTION	AUGUSTUS	8
ABINITIO_PREDICTION	Braker2	1
PROTEIN	Genome threader	1

[ii] 59,613 protein-coding genes (59,613 transcripts)

*Add 2,524 genes that were present in [ii] but absent in [i]
*Update annotation of 1,023 genes in [i] based on [ii]

Small RNA prediction with cmscan and tRNAscan
(These are not protein-coding genes but described in GFF file)

[iii] 33,829 protein-coding genes (40,363 transcripts)
3,425 non-coding genes

Gene prediction dataset	Protein BUSCO benchmark (ver3.0) Complete	Fragmented	Missing
Final dataset [iii]	1,372 (95.3%)	16	52
ONT-based gene dataset [i]	1,362 (94.6%)	24	55
EVM [ii]	1,348 (93.6%)	33	59

d

Genome reference	ONT-based gene prediction	Genome threader
Aarabidopsis thaliana TAIR10	Yes	Yes
Solanum lycopersicum iTAG3.0	Yes	Yes
Cucumis melo DHL92 CM4.0	Yes	Yes
Cucumis sativus Chineese Long ver3.0	Yes	Yes
Cucumis sativus PI183967	Yes	Yes
Cucumis sativus Gy14 ver1.0	-	Yes
Citrullus lanatus 97103 ver1.0	Yes	Yes
Lagenaria siceraria USVL1Vr-Ls	Yes	Yes
Cucurbita moschata Rifu ver1.1	Yes	Yes
Cucurbita maxima Rimu ver1.1	Yes	Yes

Supplementary Fig. 7 Summary of ONT-based gene prediction in Harukei-3

a Read-length histogram of ONT direct RNA-seq in Harukei-3. **b** Examples of RNA-seq read alignment. Reads of ONT RNA-seq (bottom) or Illumina RNA-seq (top, control) were aligned to Harukei-3 ver. 1.41 genomic sequence. Although the ONT reads contained many errors as shown by the purple lines, it was enough to predict exon-intron gene structure. **c** Summarized procedure of gene prediction in Harukei-3 ver. 1.41 genome reference. ONT-based method that integrates several software, such as Minimap2, pinfish, BLAST, and HMMER, predicted 31,306 protein-coding genes with a complete BUSCO ver. 3.0 score = 1,362 (94.6%). In parallel, Evidence Modeler (EVM) was used to integrate the results of AUGUSTUS (*ab initio* method), Braker2 (Illumina RNA-seq based method), Genome Threader (protein-based method), and StringTie (transcript annotation based on Illumina RNA-seq). The EVM-based method predicted 59,613 protein-coding genes with a complete BUSCO ver. 3.0 score = 1,348 (93.6%). This EVM-based dataset was used to update the ONT-based dataset: 2,524 genes that were absent in the ONT-based dataset but present in the EVM-based dataset were added while open reading frame (ORF) structures of 1,023 genes in the ONT-based dataset were corrected based on the EVM-based dataset. Finally, a dataset of 33,829 protein-coding genes (40,363 transcripts) were obtained (BUSCO ver. 3.0 score = 1,372 [95.3%]). **d** Summary of the genome dataset used as supporting information in gene prediction analysis.

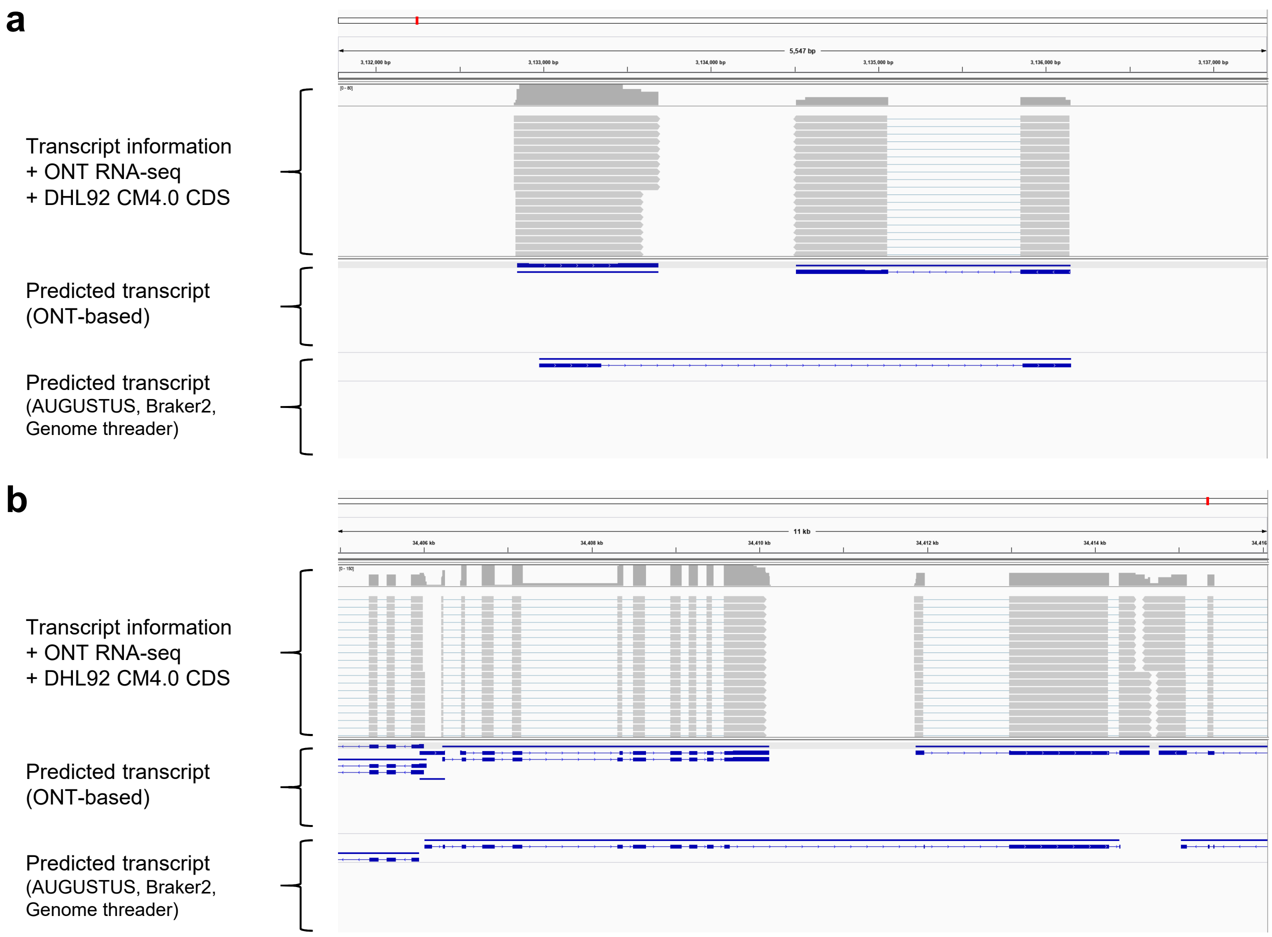

Supplementary Fig. 8 Comparison of predicted gene structures between the ONT-based method and EVM-based method
a, b Exon-intron structures of predicted genes are compared between different gene prediction methods. Top: alignment of transcript sequences obtained from ONT RNA-seq or DHL92 CM4.0 genome annotation; middle: structure of genes predicted by the ONT-based method; bottom: structure of genes predicted by EVM coupled with AUGUSTUS, Braker2, and Genome Threader. Please note that the EVM-based method predicted strange introns that were absent in the transcript sequence, whereas the ONT-based method correctly predicted exon-intron gene structure according to the transcript sequence.

a

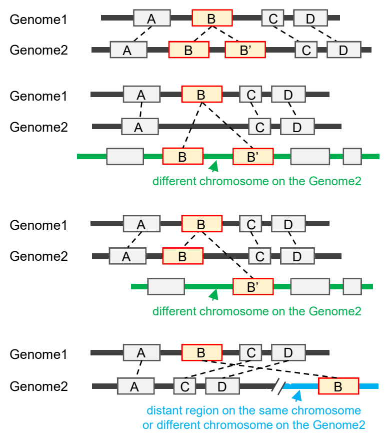

b

Enriched ID (Interproscan ID)	Fisher's exact test		Information of GO ID	Num gene
	p-value	*q*-value		
IPR012337	6.59E-150	2.70E-147	Ribonuclease H-like domain	291
IPR001584	1.92E-118	3.93E-116	Integrase | catalytic core	225
IPR005162	9.25E-100	1.26E-97	Retrotransposon gag domain	155
IPR013242	1.04E-98	1.06E-96	Retroviral aspartyl protease	171
IPR000477	3.43E-93	2.81E-91	Reverse transcriptase	178
IPR021109	1.13E-90	7.73E-89	Aspartic peptidase	170
IPR001878	1.38E-85	8.06E-84	Zinc finger | CCHC-type	152
IPR004242	1.62E-53	8.28E-52	Transposon | En/Spm-like	61
IPR023780	1.11E-49	5.04E-48	Chromo domain	113
IPR025312	1.27E-40	5.18E-39	Domain of unknown function DUF4216	40
IPR025724	2.59E-38	9.62E-37	GAG-pre-integrase domain	59
IPR013103	2.08E-36	7.09E-35	Reverse transcriptase | RNA-dependent DNA polymerase	79
IPR025452	2.44E-30	7.68E-29	Domain of unknown function DUF4218	34
IPR008906	2.28E-28	6.66E-27	HAT dimerisation domain | C-terminal	44
IPR016197	5.80E-25	1.54E-23	Chromo domain-like	51
IPR025525	6.01E-25	1.54E-23	Domain of unknown function DUF4413	31
IPR003653	1.02E-19	2.45E-18	Peptidase C48 | SUMO/Sentrin/Ubl1	32
IPR019557	1.96E-16	4.32E-15	Aminotransferase-like | plant mobile domain	32
IPR003656	2.01E-16	4.32E-15	Zinc finger | BED-type predicted	24
IPR015410	8.28E-15	1.69E-13	Domain of unknown function DUF1985	23

Supplementary Fig. 9 Retrotransposon-related functions are enriched in candidates of copy number or presence/absence polymorphisms between Harukei-3 and DHL92 genomes
a Schematic illustration of the definition of copy number polymorphism (CNP) and presence/absence polymorphism (PAP). Four cases are assumed; genes are tandemly duplicated at the same chromosome region or a different chromosome (top, 2nd from the top). Gene is apparently copied to a distant region of the same chromosome or a different chromosome (3rd from the top). Gene is apparently translocated to a distant region of the same chromosome or a different chromosome (bottom). In total, 1203 genes or protein-coding sequences were applied to these cases (also shown in Fig. 2a). **b** InterPro ID enriched in the 1203 CNP or PAP candidates. ID enrichment analysis was performed using the 'GO enrichment analysis tool' in Melonet-DB (https://melonet-db.dna.affrc.go.jp/ap/got).

a

Genome reference: Harukei-3 ver. 1.41 (var. *reticulatus*)

			Concordantly aligned		Discordantly aligned		One mate aligned	
Accession	Species, subgroup	Total reads	%aligned	%complete match	%aligned	%complete match	%aligned	%complete match
Harukei-3	*C. melo* var. *reticulatus*	459,736,006	99.18	90.68	0.37	0.33	0.08	0.03
Honey dew	*C. melo* var. *inodorus*	54,438,848	93.91	66.52	1.03	0.50	1.73	0.41
Spicy	*C. melo* var. *cantalupensis*	56,537,156	96.68	75.53	0.30	0.13	0.94	0.27
Manshuu	*C. melo* var. *makuwa*	55,690,052	88.87	42.88	1.02	0.22	3.43	0.61
Ougon-9	*C. melo* var. *makuwa*	64,043,888	88.90	43.41	1.16	0.27	3.48	0.63
JSS-6	*C. agrestis* (wild melon)	61,361,440	88.98	43.20	1.06	0.22	3.44	0.61

Genome reference: DHL92 CM3.6.1 (var. *inodorus x conomon-chinensis*)

			Concordantly aligned		Discordantly aligned		One mate aligned	
Accession	Species, subgroup	Total reads	%aligned	%complete match	%aligned	%complete match	%aligned	%complete match
Harukei-3	*C. melo* var. *reticulatus*	459,736,006	93.40	60.69	1.31	0.53	1.59	0.38
Honey dew	*C. melo* var. *inodorus*	54,438,848	88.98	51.75	2.56	0.87	3.07	0.67
Spicy	*C. melo* var. *cantalupensis*	56,537,156	87.84	45.95	2.46	0.68	3.48	0.71
Manshuu	*C. melo* var. *makuwa*	55,690,052	90.17	56.30	2.22	0.75	2.59	0.70
Ougon-9	*C. melo* var. *makuwa*	64,043,888	90.37	57.17	2.26	0.80	2.61	0.70
JSS-6	*C. agrestis* (wild melon)	61,361,440	89.70	55.52	2.45	0.84	2.76	0.75

Genome reference: Payzawat, ASM976082v1 (var. *inodorus*)

			Concordantly aligned		Discordantly aligned		One mate aligned	
Accession	Species, subgroup	Total reads	%aligned	%complete match	%aligned	%complete match	%aligned	%complete match
Harukei-3	*C. melo* var. *reticulatus*	459,736,006	93.88	69.44	0.86	0.36	1.41	0.39
Honey dew	*C. melo* var. *inodorus*	54,438,848	90.09	63.54	1.47	0.58	2.71	0.85
Spicy	*C. melo* var. *cantalupensis*	56,537,156	88.86	55.37	1.23	0.31	3.18	0.85
Manshuu	*C. melo* var. *makuwa*	55,690,052	84.23	36.96	1.50	0.26	4.67	0.84
Ougon-9	*C. melo* var. *makuwa*	64,043,888	84.20	37.48	1.62	0.31	4.70	0.85
JSS-6	*C. agrestis* (wild melon)	61,361,440	84.26	37.05	1.57	0.28	4.67	0.84

b

Genome reference: Harukei-3 ver. 1.41 (var. *reticulatus*)

Accession	Intergenic	Synonymous	Non-CDS exon	Intron	Splicing junction	Nonsense	Missense	Mutation at start codon	Mutation at stop codon*
Harukei-3	22,588	336	1,727	6,774	31	145	570	8	41
Honey dew	748,925	18,512	33,906	211,054	579	1,313	20,743	119	459
Spicy	484,305	13,176	24,174	139,551	431	1,589	17,440	92	298
Manshuu	1,658,203	38,035	68,107	423,972	1,147	2,944	45,327	246	849
Ougon-9	1,797,255	40,739	72,799	453,835	1,256	3,025	47,783	260	943
JSS-6	1,485,476	35,092	62,158	378,455	1,084	2,610	41,023	220	780

Genome reference: DHL92 CM3.6.1 + CM4.0 annotation (var. *inodorus x conomon-chinensis*)

Accession	Intergenic	Synonymous	Non-CDS exon	Intron	Splicing junction	Nonsense	Missense	Mutation at start codon	Mutation at stop codon*
Harukei-3	1,548,547	24,062	36,825	252,908	1,025	2,621	29,119	389	651
Honey dew	1,350,119	23,728	38,207	239,690	1,003	2,537	27,229	349	593
Spicy	1,616,475	29,635	46,548	297,333	1,223	3,353	35,955	421	689
Manshuu	962,558	17,714	29,345	175,515	822	2,584	22,426	299	531
Ougon-9	911,926	17,143	27,925	169,146	758	2,346	21,155	299	516
JSS-6	1,007,793	19,160	31,793	186,004	838	2,647	23,955	322	564

Supplementary Fig. 10 Evaluation of melon genome references using Illumina short read resequencing
a Comparison of short read alignment ratios between Harukei-3 (ver.1.41), DHL92[1,7] (CM3.6.1 assembly), and Payzawat[6] (ASM976082v1) genomes. Illumina short reads of six melon accession (Harukei-3, Honey Dew, Spicy, Manshuu, Ougon-9, and JSS-6) were aligned to the genome sequences with bowtie2. **b** Comparison of detected mutation type between Harukei-3 (ver. 1.41) and DHL92 (CM3.6.1 assembly + CM4.0 annotation).

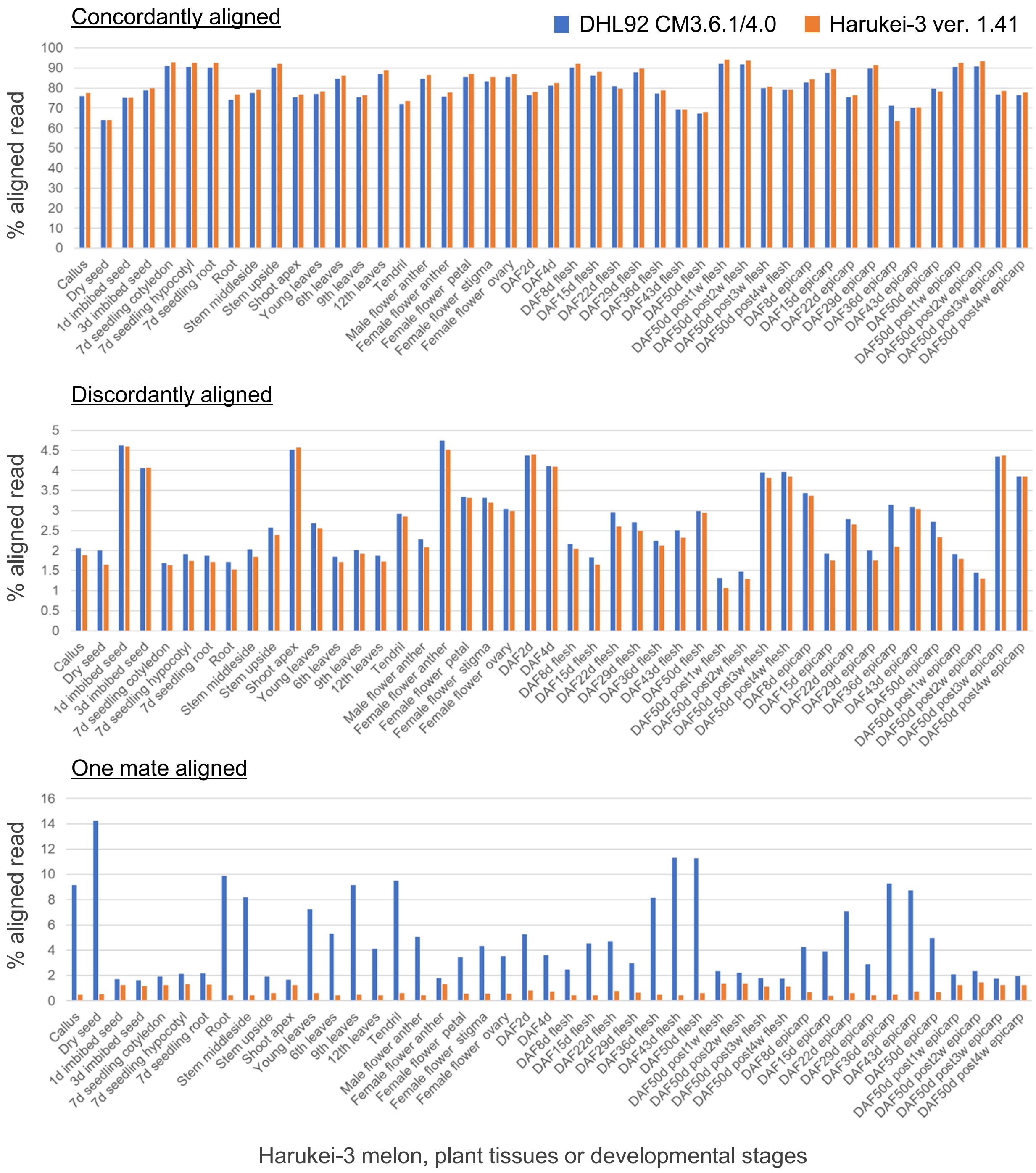

Number of genes with FPKM > 0.1 in at least one RNA-seq sample

Harukei-3 : **27,687** genes
DHL92 : **16,013** genes

Supplementary Fig. 11 Comparison of Illumina RNA-seq alignment ratio
Illumina RNA-seq short reads of 45 tissue-wide samples (also summarized in Fig. 3a) were aligned to Harukei-3 (ver. 1.41) or DHL92 (CM 3.6.1 assembly + CM 4.0 annotation) genomes with HISAT2; then, alignment ratios were compared between the two genomes. Gene expression levels were calculated with StringTie, then, the number of genes with FPKM ≥ 0.1 in at least one sample was calculated.

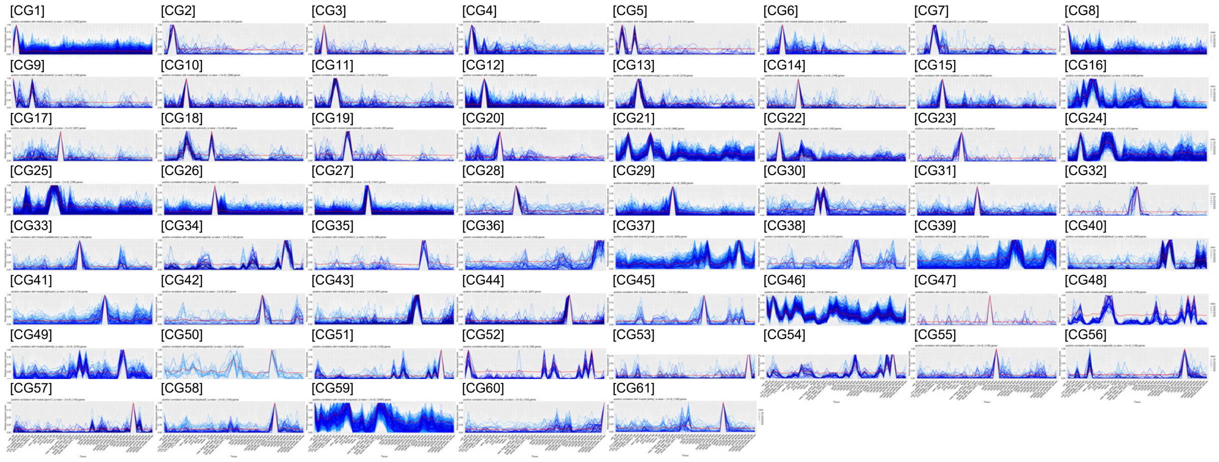

Group	Tissue specificity	positive	negative
CG1	Dry seed	1,552	0
CG2	Imbibed seed	87	0
CG3	Imbibed seed	93	0
CG4	Imbibed seed | callus	251	0
CG5	Imbibed seed | root (seedling) | callus	41	0
CG6	Hypocotyl (seedling)	271	0
CG7	Hypocotyl (seedling) | root (seedling)	92	0
CG8	Callus	844	0
CG9	Root (seedling) | callus | root | imbibed seed	106	0
CG10	Root | root (seedling)	298	0
CG11	Root | root (seedling)	179	0
CG12	Root | root (seedling)	954	0
CG13	Root | stem | root (seedling) | hypocotyl(seedling)	219	0
CG14	Shoot apex	148	0
CG15	Stem	292	0
CG16	Stem | root | hypocotyl (seedling)	326	0
CG17	Tendril	227	0
CG18	Tendril | root | stem | hypocotyl (seedling) | root (seedling)	84	0
CG19	Young leaves | shoot apex	83	0
CG20	Young leaves | shoot apex | cotyledon (seedling)	134	0
CG21	Cotyledon (seedling) | young leaves	486	10
CG22	Cotyledon (seedling) | young leaves | expanded leaves	163	0
CG23	Expanded leaves | tendril	70	0
CG24	Expanded leaves | young leaves | cotyledon (seedling)	411	28
CG25	Expanded leaves | young leaves | cotyledon (seedling) | tendril | hypocotyl (seedling)	789	18
CG26	Male anther	771	0
CG27	Male anther | female anther	1,641	17
CG28	Male anther | female anther | petal | stigma	128	3
CG29	Petal	562	0
CG30	Petal | stigma | female anther | male anther	147	0
CG31	Stigma	341	0

Group	Tissue specificity	positive	negative
CG32	Ovary at DAF0, 2, 4	58	0
CG33	Ovary at DAF0, 2, 4 | fruit epicarp DAF8, 15	166	0
CG34	Fruit epicarp around DAF36 to 50 (highest at DAF36)	142	1
CG35	Fruit epicarp around DAF8 to 15 (highest at DAF15)	89	0
CG36	Fruit epicarp post harvest 1w-4w (highest at 2w)	154	0
CG37	Fruit flesh and epicarp around DAF22 to 50	850	14
CG38	Fruit flesh and epicarp around DAF36 to 50 (highest at DAF43)	131	1
CG39	Fruit flesh and epicarp post harvest 1w-4w (highest at 1w)	645	10
CG40	Fruit flesh and epicarp post harvest 1w-4w (highest at 3w)	385	25
CG41	Fruit flesh around DAF36 to 50 (highest at DAF50) | post harvest 1w	418	2
CG42	Fruit flesh post harvest 1w-4w (highest at 2w)	67	0
CG43	Fruit flesh post harvest 1w-4w (highest at 3w)	481	1
CG44	Fruit flesh post harvest 3w-4w (highest at 4w)	267	0
CG45	-	99	0
CG46	-	804	7
CG47	-	43	0
CG48	-	105	2
CG49	-	276	0
CG50	-	46	0
CG51	-	120	0
CG52	-	56	0
CG53	-	123	0
CG54	-	63	0
CG55	-	136	0
CG56	-	139	0
CG57	-	143	0
CG58	-	144	0
CG59	-	3,525	23
CG60	-	153	0
CG61	-	194	0
CG62	-	0	0

Supplementary Fig. 12 WGCNA clustering of tissue-wide transcriptome dataset

WGCNA co-expression clustering was performed based on the tissue-wide transcriptome dataset of Harukei-3 (Fig. 3a). In total, 62 co-expression groups were found. Pink and red rectangles indicate co-expression groups associating with fruit development or post-harvest ripening fruits, respectively.

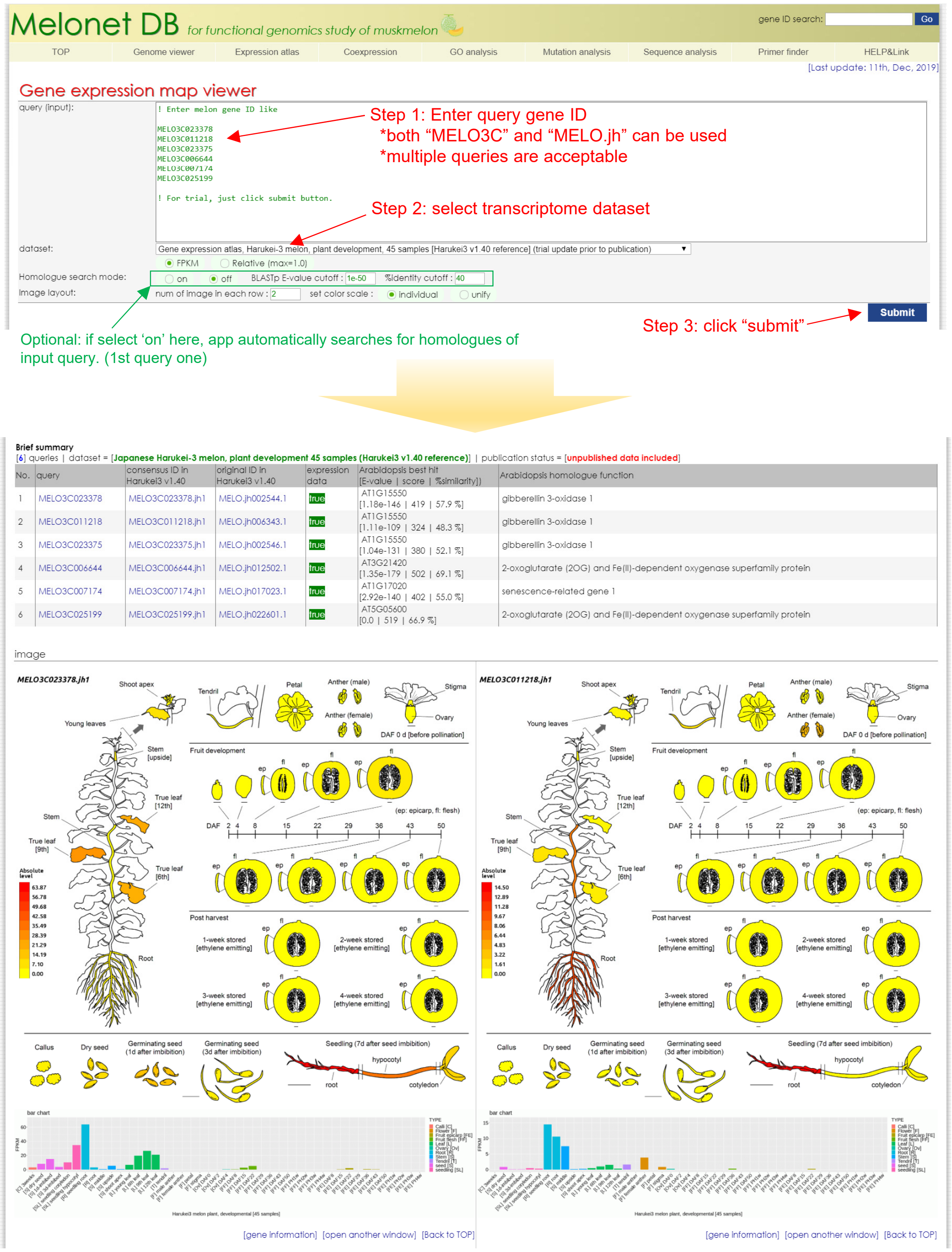

Supplementary Fig. 13 Updated version of 'Gene expression map viewer' in the Melonet-DB
This web application is available at the following URL: https://melonet-db.dna.affrc.go.jp/ap/mvw.

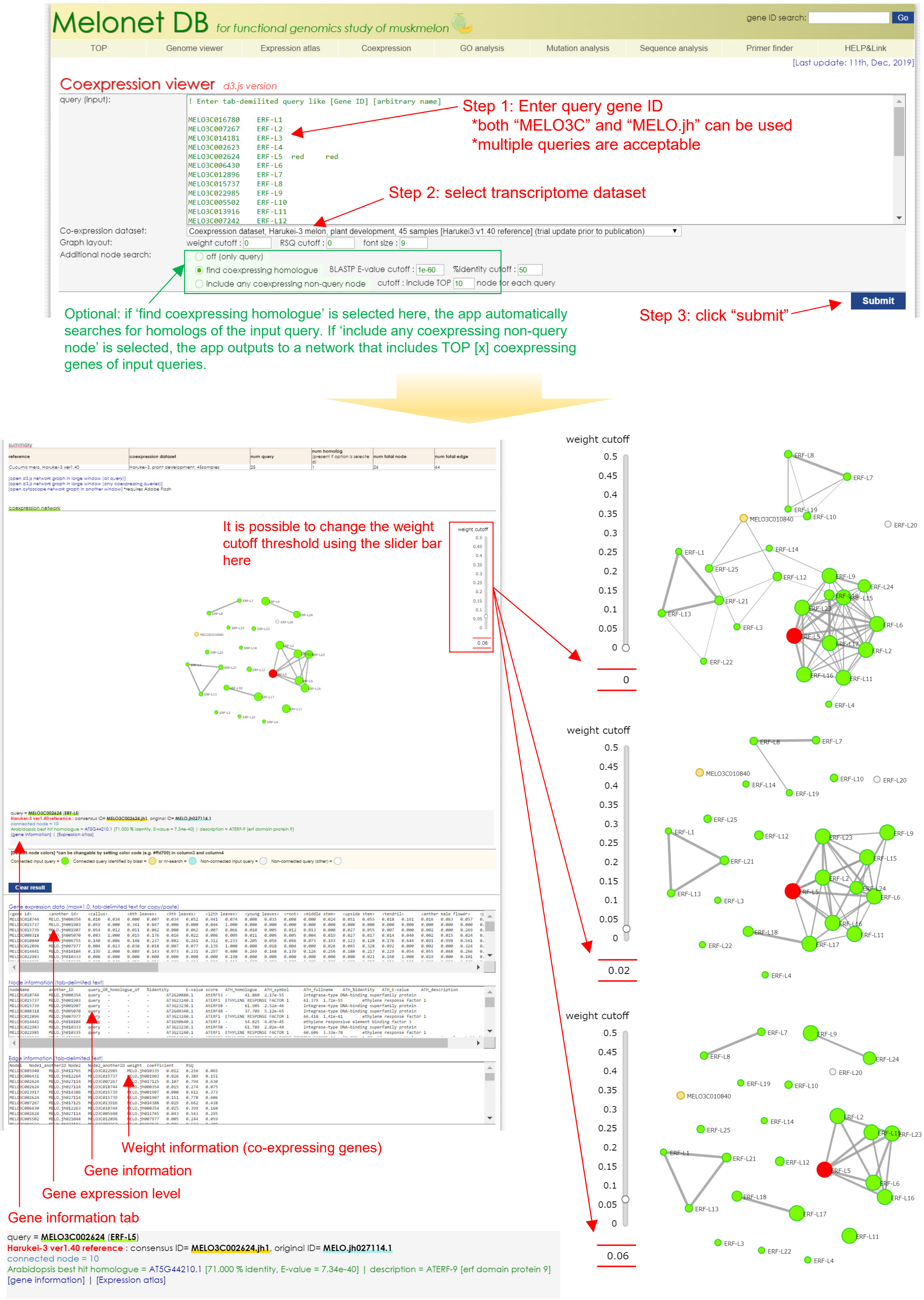

Supplementary Fig. 14 Updated version of 'Coexpression viewer' in the Melonet-DB
This web application is available at the following URL: https://melonet-db.dna.affrc.go.jp/ap/mds.

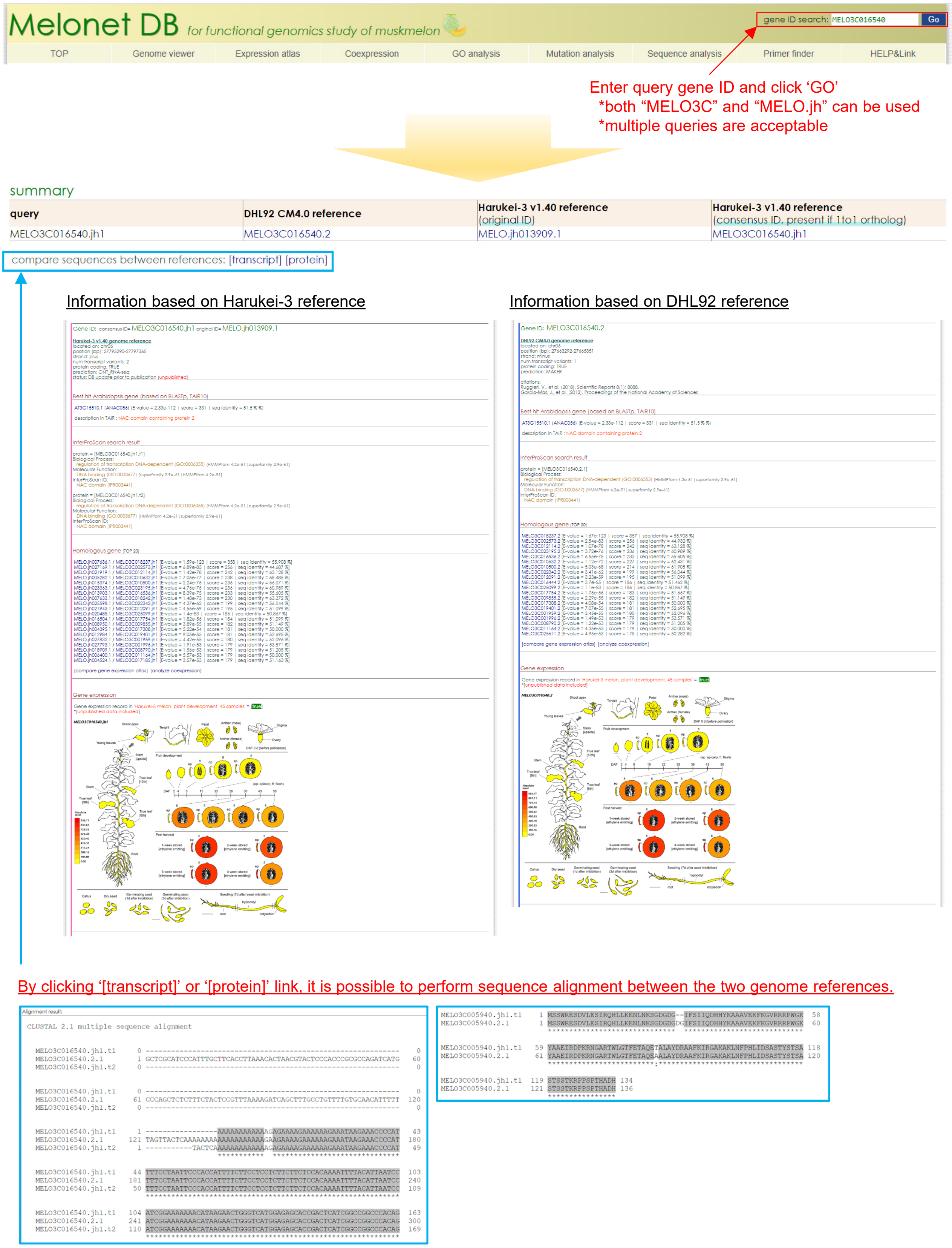

Supplementary Fig. 15 Gene information search function in the Melonet-DB
This web application is available in Melonet-DB web site: https://melonet-db.dna.affrc.go.jp/.

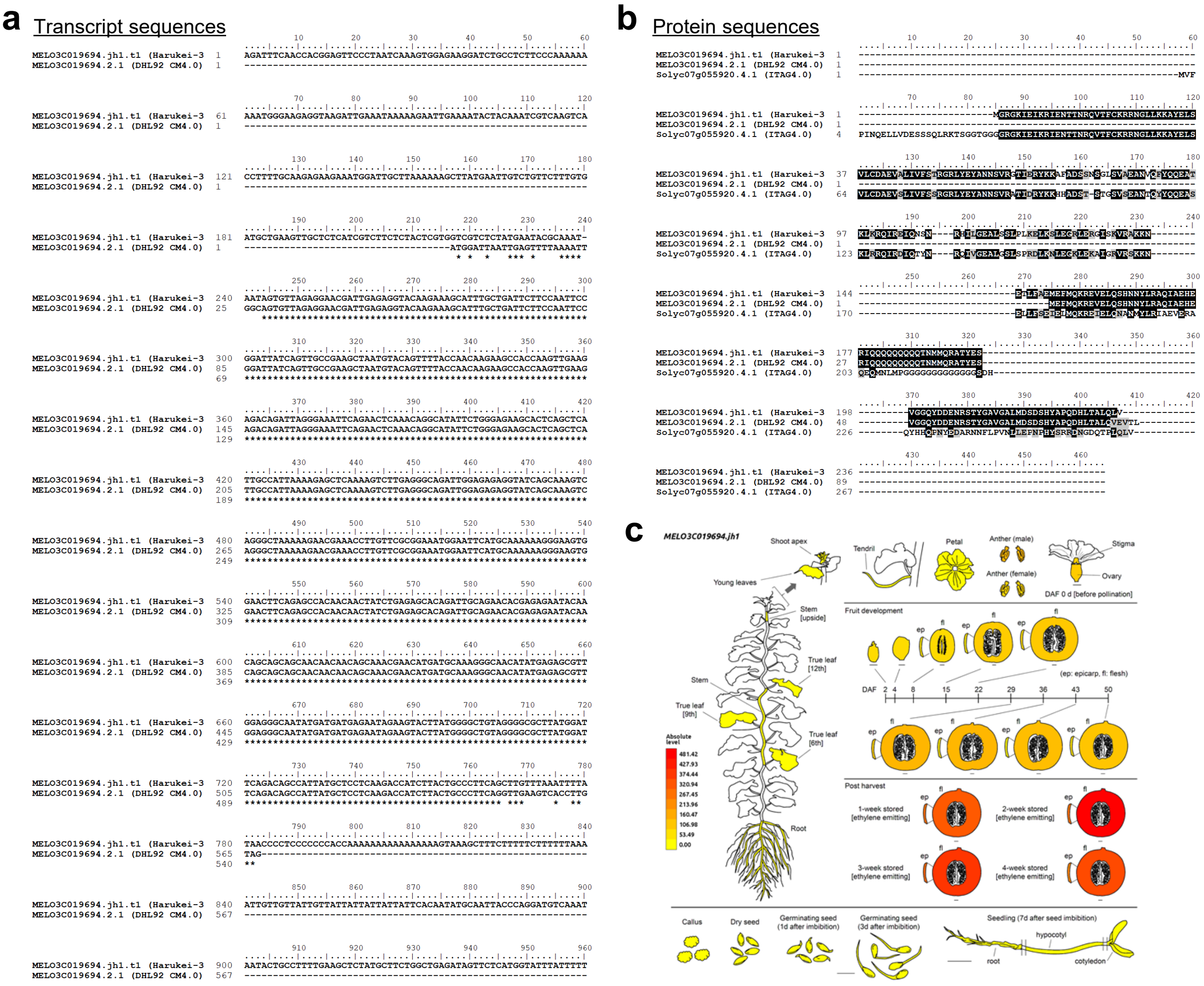

Supplementary Fig. 16 Sequence and gene expression pattern of *MELO3C019694*

a, b Comparison of transcript (a) or protein amino acid (b) sequences of an *AGAMOUS*-like gene, *MELO3C019694*, between Harukei-3 (top) and DHL92 (bottom). c Tissue-wide gene expression pattern of *MELO3C019694* in Harukei-3.

a

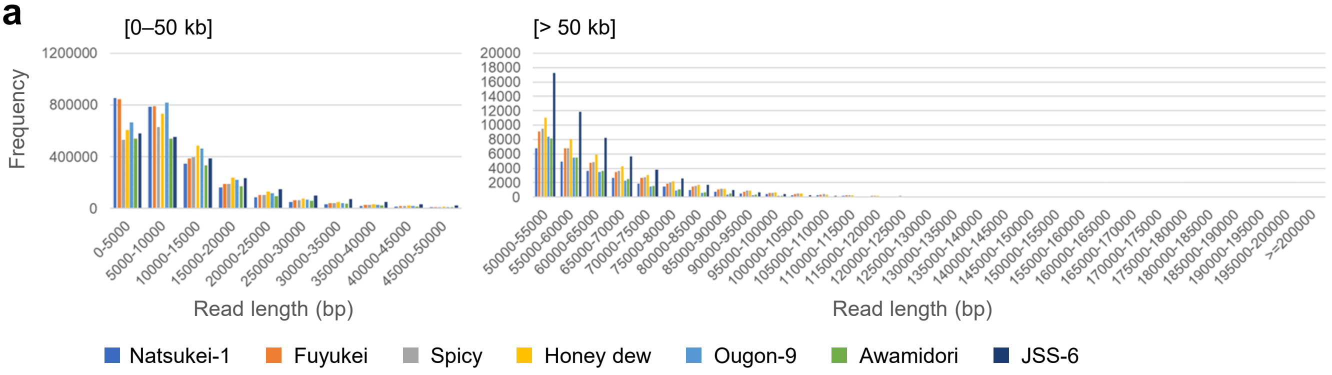

b

Accession	Read count	Total length (bp)	Average (bp)	Max (bp)	N_{50} (bp)
Natsukei1	2,393,835	22,828,785,451	9,536	218,124	13,972
Fuyukei	2,519,061	26,060,465,229	10,345	313,129	15,542
Spicy	2,060,998	24,591,127,917	11,932	286,854	16,856
HoneyDew	2,444,716	29,608,876,134	12,111	244,739	16,941
Ougon9	2,490,216	27,243,224,015	10,940	204,102	15,172
Awamidori	1,867,169	21,287,972,918	11,401	218,625	16,795
JSS6	2,248,973	30,902,529,697	13,741	207,635	21,374

c Canu ver. 1.8 contig assembly

Accession	Num contig	Total length (Mb)	Average (kb)	Max (kb)	N_{50} (kb)
Natsukei-1	367	371.8	1,013	13,823	3,946
Fuyukei	265	371.3	1,401	20,607	4,863
Spicy	223	367.9	1,650	20,683	5,966
HoneyDew	288	373.2	1,296	15,114	5,084
Ougon-9	250	361.0	1,444	19,751	4,143
Awamidori	271	361.0	1,332	16,057	5,513
JSS6	129	361.6	2,803	32,807	10,243

Corrected contig assembly

Accession	Num contig	Total length (Mb)	Average (kb)	Max (kb)	N_{50} (kb)
Natsukei-1	462	373.7	809	13,898	3,505
Fuyukei	349	373.1	1,069	18,205	4,043
Spicy	262	369.6	1,411	20,815	5,044
HoneyDew	354	374.3	1,057	11,987	4,767
Ougon-9	295	363.1	1,231	17,961	3,706
Awamidori	315	362.8	1,152	16,150	4,709
JSS6	157	363.6	2,316	25,717	10,148

Supplementary Fig. 17 Contig assembly in seven melon accessions
a Histograms of sequenced reads in Oxford NanoPore technology (ONT; R9.4.1 flow cell). **b** Read length statistics of ONT reads. **c** Sequence length statistics of the contig assemblies in seven melon accessions. Contigs were initially assembled with Canu ver. 1.8 software based on ONT reads and the resultant contigs were subjected to racon and medaka polishing to correct sequence errors. Then, contigs with chimeric sequences (incorrectly assembled contigs) were identified and split by searching for the positions where the number of aligned ONT reads were less than four. The top panel indicates the value of the sequence length statistics of the Canu ver. 1.8 contig assemblies while the bottom indicates those of the corrected contig assemblies.

References

1. Garcia-Mas, J. *et al.* The genome of melon (*Cucumis melo* L.). *Proc Natl Acad Sci* **109** (2012).
2. Kawazu, Y. *et al.* QTL mapping for resistance to Cucurbit chlorotic yellows virus in melon (*Cucumis melo* L.). *Euphytica* **214**, 239 (2018).
3. Argyris, J.M. *et al.* Use of targeted SNP selection for an improved anchoring of the melon (*Cucumis melo* L.) scaffold genome assembly. *BMC Genomics* **16**, 014-1196 (2015).
4. Zheng, Y. *et al.* Cucurbit Genomics Database (CuGenDB): a central portal for comparative and functional genomics of cucurbit crops. *Nucleic Acids Research* **47**, D1128-D1136 (2018).
5. Diaz, A. *et al.* Anchoring the consensus ICuGI genetic map to the melon (Cucumis melo L.) genome. *Molecular Breeding* **35**, 188 (2015).
6. Zhang, H. *et al.* A High-Quality Melon Genome Assembly Provides Insights into Genetic Basis of Fruit Trait Improvement. *iScience* **22**, 16-27 (2019).
7. Ruggieri, V. *et al.* An improved assembly and annotation of the melon (*Cucumis melo* L.) reference genome. *Scientific Reports* **8**, 8088 (2018).
