# Supplementary material for: Comparative genomics of muskmelon reveals a potential role for retrotransposons in the modification of gene expression
Source: Commun Biol. 2020 Aug 13;3:432. doi: 10.1038/s42003-020-01172-0 (PMC7426833; doi:10.1038/s42003-020-01172-0)
Supplement: Supplementary file 11 — Reporting Summary [file 42003_2020_1172_MOESM11_ESM.pdf]

nature research

Corresponding author(s): Hiroshi Ezura, Ryoichi Yano

Last updated by author(s): Jul 10, 2020

Reporting Summary

Nature Research wishes to improve the reproducibility of the work that we publish. This form provides structure for consistency and transparency in reporting. For further information on Nature Research policies, see Authors & Referees and the Editorial Policy Checklist.

Statistics

For all statistical analyses, confirm that the following items are present in the figure legend, table legend, main text, or Methods section.

n/a	Confirmed	
☐	☒	The exact sample size (*n*) for each experimental group/condition, given as a discrete number and unit of measurement
☐	☒	A statement on whether measurements were taken from distinct samples or whether the same sample was measured repeatedly
☐	☒	The statistical test(s) used AND whether they are one- or two-sided<br>*Only common tests should be described solely by name; describe more complex techniques in the Methods section.*
☐	☒	A description of all covariates tested
☐	☒	A description of any assumptions or corrections, such as tests of normality and adjustment for multiple comparisons
☐	☒	A full description of the statistical parameters including central tendency (e.g. means) or other basic estimates (e.g. regression coefficient) AND variation (e.g. standard deviation) or associated estimates of uncertainty (e.g. confidence intervals)
☐	☒	For null hypothesis testing, the test statistic (e.g. *F*, *t*, *r*) with confidence intervals, effect sizes, degrees of freedom and *P* value noted<br>*Give P values as exact values whenever suitable.*
☐	☒	For Bayesian analysis, information on the choice of priors and Markov chain Monte Carlo settings
☐	☒	For hierarchical and complex designs, identification of the appropriate level for tests and full reporting of outcomes
☐	☒	Estimates of effect sizes (e.g. Cohen's *d*, Pearson's *r*), indicating how they were calculated

Our web collection on statistics for biologists contains articles on many of the points above.

Software and code

Policy information about availability of computer code

Data collection	We used Oxford nanopore technology (ONT) Minknow software release 19 to obtain raw ONT sequencing data (fast5 files).
Data analysis	In this study, we used the following software; ONT guppy version 3.2.1 and 3.3.0, Canu version 1.8, IrysSolve version 3.2, SSPACE version 3.0, minimap2, gffread, Pinfish, hmmsearch, NCBI blast version 2.40+, AUGUSTUS version 3.3.2, BRAKER2, genome threader version 1.7.0, Evidence modeler version 1.1, RepeatModeler, RepeatMasker, Hisat2, StringTie, bowtie2, Genome analysis tool kit, LAST, R version 3.2.3, Circos, Interproscan. All of these software are publicly available.

For manuscripts utilizing custom algorithms or software that are central to the research but not yet described in published literature, software must be made available to editors/reviewers. We strongly encourage code deposition in a community repository (e.g. GitHub). See the Nature Research guidelines for submitting code & software for further information.

Data

Policy information about availability of data

All manuscripts must include a data availability statement. This statement should provide the following information, where applicable:

- Accession codes, unique identifiers, or web links for publicly available datasets
- A list of figures that have associated raw data
- A description of any restrictions on data availability

Raw sequencing data used in this study can be found in the NCBI database under the following Bioproject accession numbers: PRJNA603155 (genome sequencing dataset of Harukei-3 melon), PRJNA603146 (ONT cDNA RNA-seq), PRJNA603129 (ONT direct RNA-seq), PRJNA624817 (ONT genome sequencing dataset of seven melon accessions), PRJNA603204 (tissue-wide RNA-seq of Harukei-3 melon), or PRJNA603202 (leaf RNA-seq in the greenhouse). Genome assembly and annotation of Harukei-3 melon (ver1.40 genome reference) is available on Melonet-DB (https://melonet-db.dna.affrc.go.jp/ap/dnl). All of these dataset can be downloaded and used without any restrictions.

Field-specific reporting

Please select the one below that is the best fit for your research. If you are not sure, read the appropriate sections before making your selection.

☒ Life sciences ☐ Behavioural & social sciences ☐ Ecological, evolutionary & environmental sciences

For a reference copy of the document with all sections, see nature.com/documents/nr-reporting-summary-flat.pdf

Life sciences study design

All studies must disclose on these points even when the disclosure is negative.

Sample size	For de novo genome assembly of Harukei-3 melon, we obtained 32.6 Gb of Oxford nanopore technology (ONT) sequencing data, 19.5 Gb of PacBio RS II data, 37 Gb of Illumina Hiseq paied-end data, 74 Gb of Illumina mate pair data, 86 Gb of BioNano optical map data. For de novo genome assembly (contig assembly) of seven additional melon accessions, we obtained 21.1 to 30.9 Gb of ONT sequencing data. For tissue-wide transcriptome and co-expression study, we used RNA-seq data that were obtained from 45 tissue-wide samples. This dataset covers most melon tissues, including roots, stems, leaves, seedlings, germinating seeds, and flower organs, as well as fruit developmental stages from pollination to ripening. For field transcriptome study in the greenhouse conditions, we obtained RNA-seq data in 75 samples from 18 independent plants.
Data exclusions	No data exlusion
Replication	In tissue-wide transcriptome study, we used RNA-seq data from 45 samples including 24 fruit samples. We obtained at least three RNA-seq data in each of the following fruit development stages; early (Day after fertilization [DAF] 2-8), mid (DAF 15-29), maturation (DAF 36-50), and ripening (post harvest 1-4 weeks). For field transcriptome study in the greenhouse conditions, we obtained and used 75 RNA-seq samples from 18 independent plants.
Randomization	In tissue-wide transcriptome study, samples were divided into groups according to the tissue type or developmental stage. In filed transcriptome study in the greenhouse conditions, samples were divided into group according to the sampling date.
Blinding	In this study, we did not perform analysis that requires blinding method.

Reporting for specific materials, systems and methods

We require information from authors about some types of materials, experimental systems and methods used in many studies. Here, indicate whether each material, system or method listed is relevant to your study. If you are not sure if a list item applies to your research, read the appropriate section before selecting a response.

Materials & experimental systems

n/a	Involved in the study
☒	☐ Antibodies
☒	☐ Eukaryotic cell lines
☒	☐ Palaeontology
☒	☐ Animals and other organisms
☒	☐ Human research participants
☒	☐ Clinical data

Methods

n/a	Involved in the study
☒	☐ ChIP-seq
☒	☐ Flow cytometry
☒	☐ MRI-based neuroimaging
